# Discovery of Novel SARS-CoV-2 Fusion Inhibitors—Posaconazole-Polyarginine Conjugates

**DOI:** 10.3390/v18070737

**Published:** 2026-07-02

**Authors:** Yihui Jin, Lili Qu, Xin Gao, Xiao Qi, Dongmin Zhao, Lu Ga, Yan Zhao, Guodong Liang, Yunfeng Xiao, Yuheng Ma

**Affiliations:** 1 Key Laboratory for Candidate Drug Design and Screening Based on Chemical Biology, College of Pharmacy, Inner Mongolia Medical University, Hohhot 010110, China; 2 Department of Pharmacy, The Affiliated Hospital of Inner Mongolia Medical University, Hohhot 010030, China; 3 State Key Laboratory of Natural and Biomimetic Drugs, Peking University, Beijing 100191, China; 4 Peptide Drugs Research and Development Center, Zhen-Xiang Technology Co., Ltd., Hohhot 011500, China

**Keywords:** SARS-CoV-2, fusion inhibitors, posaconazole, polyarginine, conjugates

## Abstract

**Objectives:** The ongoing evolution of severe acute respiratory syndrome coronavirus 2 (SARS-CoV-2) and the current treatment limitations—particularly the emergence of drug resistance and the reduced efficacy of some existing drugs against new variants—highlight the need for novel antiviral strategies with novel action mechanisms. Fusion inhibitors that disrupt six-helix bundle (6-HB) formation during viral entry represent a promising approach. Posaconazole, an antifungal agent, has been identified as a weak fusion inhibitor, but suffers from poor membrane permeability and modest activity. This study aimed to enhance its antiviral potency by conjugating it with cell-penetrating polyarginine peptides and to investigate the mechanism of action. **Methods:** A series of posaconazole-polyarginine conjugates were synthesized via click chemistry. Antiviral activity was evaluated using pseudotyped SARS-CoV-2 Omicron XDV in HEK293T cells. Mechanisms were investigated by circular dichroism, native PAGE, size-exclusion HPLC, molecular docking, and isothermal titration calorimetry. Metabolic stability was assessed using hepatic microsomes. **Results:** Posa-R8 exhibited potent antiviral activity comparable to the clinical candidate EK1, with minimal cytotoxicity. Mechanistic studies confirmed that Posa-R8 binds the HR2 region of the spike protein, disrupts 6-HB formation, and inhibits membrane fusion. It also showed strong lipid bilayer affinity and improved phase I metabolic stability over EK1. **Conclusions:** Polyarginine conjugation enhances the membrane-binding affinity and antiviral efficacy of posaconazole. Posa-R8 represents a promising lead for developing next-generation SARS-CoV-2 fusion inhibitors.

## 1. Introduction

Coronaviruses are a large family of enveloped, positive-sense single-stranded RNA viruses that infect a broad range of hosts, including humans [1]. Currently, seven coronaviruses are known to cause disease in humans [2]. Among these, HCoV-229E, HCoV-OC43, HCoV-NL63, and HCoV-HKU1 exhibit low pathogenicity and typically cause mild upper respiratory symptoms [3]. In contrast, SARS-CoV-2, Severe Acute Respiratory Syndrome Coronavirus (SARS-CoV), and Middle East Respiratory Syndrome Coronavirus (MERS-CoV) can invade the lower respiratory tract, leading to severe pneumonia and posing significant threats to human health [4]. Notably, coronavirus disease 2019 (COVID-19), caused by SARS-CoV-2, has had a profound impact on global public health and healthcare systems. According to the World Health Organization (WHO), as of April 2026, over 779 million confirmed COVID-19 cases and more than 7.11 million deaths have been reported worldwide. The high mutation rate of SARS-CoV-2 has driven the emergence of multiple variants, including Alpha, Beta, Gamma, Delta, and Omicron. Of these, the Omicron variant stands out as the most heavily mutated strain, exhibiting high transmissibility, low pathogenicity, and the ability to evade immunity [5]. Data from the Chinese Center for Disease Control and Prevention (CDC) indicate that Omicron sublineages have continuously evolved in China throughout 2025—from BA.5.2 and BF.7 early in the year to JN.1 and XDV by mid-year, and eventually to NB.1.8.1 by year-end. Obviously, in the post-pandemic era, SARS-CoV-2 is expected to coexist with humans long-term, and the continuous emergence of new variants remains a persistent global health challenge [6]. Therefore, the development of antiviral agents will remain critical for managing SARS-CoV-2 infections and may inform strategies for future coronavirus outbreaks.

The SARS-CoV-2 structural proteins include the spike protein (S), envelope protein (E), membrane protein (M), and nucleocapsid protein (N) [7]. The S protein is the primary functional protein for viral entry into host cells, consisting of S1 and S2 subunits [8]. The S1 subunit comprises an N-terminal domain (NTD) and a receptor-binding domain (RBD), which recognize and bind to angiotensin-converting enzyme 2 (ACE2) on the host–cell surface. The S2 subunit contains a fusion peptide (FP) and two heptad repeat regions (HR1 and HR2), which adopt typical α-helical structures and mediate the fusion between the viral envelope and host–cell membranes [4]. After the S1 subunit binds to ACE2 and triggers allosteric activation, the S2 subunit undergoes conformational changes, allowing the FP to insert into the host membrane. This extended pre-hairpin structure then refolds: HR1 assembles into a trimeric core, while HR2 packs antiparallel into the HR1 grooves, forming a stable six-helix bundle (6-HB) [9]. The energy released during 6-HB formation drives membrane fusion, enabling viral genetic material to enter the host–cell. So, the 6-HB is a conserved structural element essential for viral entry and has become a key target for fusion inhibitors [10]. Enfuvirtide (T20), which exerts its effect by disrupting the 6-HB conformational state in the HIV-1 envelope protein, was the first fusion inhibitor approved for clinical use in 2003 [11]. Subsequently, several HIV-1 fusion inhibitors have been developed, including Albuvirtide, approved in China in 2018, and Sifuvirtide, currently in Phase III clinical trials [12]. The success of these inhibitors provides a model for the development of antivirals targeting other viruses. SARS-CoV-2 and HIV-1 share similar membrane fusion mechanisms; therefore, targeting 6-HB to block SARS-CoV-2 membrane fusion is an effective strategy for inhibitor discovery [13]. In recent years, a number of peptide-based SARS-CoV-2 fusion inhibitors targeting the 6-HB have been reported, including the pan-coronavirus lipopeptide inhibitor CGM23, which binds to the highly conserved HR1 domain and exhibits potent broad-spectrum activity against diverse human coronaviruses both in vitro and in vivo [14].

In 2022, Mondal et al. identified an evolutionarily conserved “Ex3Lx6L” motif (x3 denotes three amino acid residues and x6 denotes six amino acid residues) in the HR2 region of coronaviruses, which plays a critical role in stabilizing the post-fusion 6-HB conformation. Via molecular docking screening, they found that Posaconazole (Posa), an FDA-approved antifungal drug, could bind to “Ex3Lx6L” motif and inhibit SARS-CoV-2 infection [15]. However, posaconazole exhibits an inhibitory activity of only 3.37 µM against SARS-CoV-2 S pseudotyped viruses, which is far lower than that of EK1, a widely used positive control in fusion inhibitor studies that disrupts SARS-CoV-2 S 6-HB formation and has demonstrated potent antiviral activity against multiple coronaviruses in preclinical models [2]. Furthermore, its poor membrane-binding affinity limits Posa ability to effectively reach the target site. To address these limitations, we hypothesized that conjugating posaconazole with cell-penetrating polyarginine peptides would enhance its membrane-binding affinity and thereby improve its antiviral efficacy.

In this study, we synthesized a series of conjugates by linking Posa with polyarginine via click chemistry; among these, Posa-R8 demonstrated anti-SARS-CoV-2 activity that was significantly stronger than Posa, but comparable to the positive control EK1. Mechanistic studies revealed that posaconazole-polyarginine conjugates can target the HR2 region to disrupt 6-HB formation, thereby inhibiting the SARS-CoV-2 membrane fusion process. Moreover, Posa-R8 displayed enhanced metabolic stability versus EK1. These structurally novel conjugates, with high activity and well-defined mechanisms, provide valuable leads for the SARS-CoV-2 therapeutics development.

## 2. Design

The 6-HB is not only a core structural element in SARS-CoV-2 membrane fusion but also a key target for fusion inhibitor development. Given that Posa exhibits lower antiviral activity than the clinical candidate EK1 and the mechanism against SARS-CoV-2 has not yet been fully elucidated [16], structurally modifying Posa to enhance efficacy and investigating how to block the SARS-CoV-2 membrane fusion process are of significant research value.

**Figure 1:**
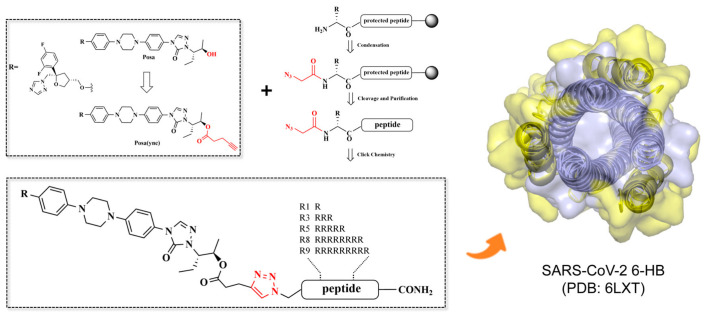
Schematic illustration of Posaconazole-polyarginine conjugates design.

Na et al. conjugated polyarginine to a triterpenoid saponin with poor bioavailability and weak antibacterial activity via click chemistry, resulting in conjugates with enhanced potent antibacterial effects [17]. Hegde et al. synthesized polyarginine-aminoglycoside conjugates by coupling neomycin and Neamine with polyarginine 6-mers and 9-mers. The conjugate effectively inhibit infection by HIV-1 X4-type strain, with the mechanism involving binding to the CXCR4 receptor to competitively inhibit the binding of the HIV-1 envelope glycoprotein gp120 to the receptor, thereby blocking viral entry into host cells [18]. These findings suggest that polyarginine modification can confer synergistic bioactivity to small molecules [19,20]. Accordingly, we covalently linked Posa to polyarginine chains of varying lengths via click chemistry (Figure 1): the N-terminal amino group of polyarginine was reacted with azidoacetic acid to introduce an azide group, while the terminal hydroxyl group of Posa was esterified with 4-pentynoic acid to introduce an alkyne. Under Cu(I) catalysis, the azide and alkyne underwent cycloaddition to form a triazole-linked conjugate, yielding Posa-R*_n_* (*n* = 1, 3, 5, 8, 9).

## 3. Materials and Methods

### 3.1. Synthesis of Posa(yne)

Posaconazole (0.7 g, 1 mmol) and pentynoic acid (0.13 g, 1.3 mmol) were dissolved in 20 mL N,N-dimethylformamide (DMF, J&K Scientific, Beijing, China). Subsequently, dicyclohexylcarbodiimide (DCC, GL Biochem, Shanghai, China; 1.24 g, 6 mmol) and 4-dimethylaminopyridine (DMAP, J&K Scientific, Beijing, China; 0.12 g, 1 mmol) were added sequentially. The mixture was heated at 40 °C for 12 h. The reaction was monitored via TLC. Upon completion, saturated saline (200 mL) was added, then the mixture was extracted with ethyl acetate (EA, J&K Scientific, Beijing, China; 50 mL × 3). Combined organic phases were dried over anhydrous magnesium sulfate, concentrated under reduced pressure, and purified by silica gel column chromatography (eluent: dichloromethane (DCM, J&K Scientific, Beijing, China)/methanol (MeOH, J&K Scientific, Beijing, China) = 20:1), yielding 536 mg of a white solid in 64% yield. ^1^H NMR (600 MHz, DMSO-d_6_) *δ* 8.34 (s, 1H), 8.32 (s, 1H), 7.78 (s, 1H), 7.48 (d, *J* = 9.0 Hz, 2H), 7.32–7.26 (m, 2H), 7.10 (d, *J* = 9.2 Hz, 2H), 6.99 (td, *J* = 8.5, 2.6 Hz, 1H), 6.94 (d, *J* = 9.0 Hz, 2H), 6.81 (d, *J* = 9.0 Hz, 2H), 5.08–5.03 (m, 1H), 4.58 (q, *J* = 14.5 Hz, 2H), 4.08 (ddd, *J* = 14.5 Hz, 2H), 7.32–7.26 (m, 2H), 7.32–7.26 (m, 2H), 7.32–7.26 (m, 2H) 4.08 (ddd, *J* = 11.3, 7.6, 4.2 Hz, 1H), 4.03 (dd, *J* = 8.7, 7.2 Hz, 1H), 3.75 (dd, *J* = 12.2, 9.1, 6.4 Hz, 2H), 3.67 (dd, *J* = 9.4, 7.6 Hz, 1H), 3.32 (s, 2H), 3.31 (s, 2H), 3.16 (s, 2H), 3.16 (s, 2H), 3.16 (s, 2H), 3.31 (s, 2H), 3.16 (s, 2H) 3.16 (dd, *J* = 6.4, 3.7 Hz, 4H), 2.73 (t, *J* = 2.5 Hz, 1H), 2.55–2.51 (m, 1H), 2.40 (d, *J* = 7.5 Hz, 1H), 2.39–2.35 (m, 2H), 2.33 (d, *J* = 2.3 Hz, 1H), 2.32–2.30 (m, 1H), 2.14 (dd, *J* = 13.2, 8.1 Hz, 1H), 1.81–1.70 (m, 2H), 1.23 (d, *J* = 6.3 Hz, 3H), 0.78 (t, *J* = 7.3 Hz, 3H). ^13^C NMR (151 MHz, CDCl_3_) *δ* 171.18, 170.84, 161.96, 159.89, 158.25, 153.08, 151.16, 144.62, 134.36,128.68, 123.50, 118.57, 116.76, 115.20, 111.44, 111.30, 104.84, 104.67, 104.50, 84.10, 84.07, 82.40, 71.50, 70.79, 69.03, 68.97, 60.40, 60.21, 55.95, 53.43, 50.67, 49.21, 38.86, 37.44, 33.60, 29.70, 22.33, 21.06, 22.33, 21.06, 29.70 22.33, 21.06, 17.31, 14.39, 14.20, 10.45. MS (*m*/*z*): calcd for C_42_H_47_F_2_N_8_O_5_, 781.36; MALDI-TOF-MS (*m*/*z*): 781.30.

### 3.2. Synthesis of Peptides

We employed the Fmoc (9-fluorenylmethoxycarbonyl)-based solid-phase peptide synthesis (SPPS) strategy to synthesize peptides. The Fmoc-protected amino acids were acquired from Myriad (Shanghai, China). The Rink-Amide resins with a loading capacity of 0.53 mmol/g were purchased from Sunresin New MATERIALS Co., Ltd. (Xi’an, China). During the synthesis, we alternately coupled each amino acid onto the resin using N,N′-Diisopropylcarbodiimide (DIC, GL Biochem, Shanghai, China) and 1-hydroxybenzotriazole (HOBT, GL Biochem, Shanghai, China). Subsequently, the Fmoc-protected group was removed with a 20% 4-methylpyridine/N,N-dimethylformamide (DMF, GL Biochem, Shanghai, China) solution. After each amino acid coupling reaction or Fmoc deprotection reaction, the resin was washed twice with DMF and dichloromethane (DCM). To achieve amino-terminal acetylation of the peptides, a mixed solution of acetic anhydride and N,N-Diisopropylethylamine (DIEA, J&K Scientific, Beijing, China) (*v*:*v* = 4:1) was added to the resin. The final molecular weight (M.W.) of the purified peptides was confirmed by MALDI-TOF-MS (Autoflex III, Bruker Daltonics, Billerica, MA, USA).

### 3.3. Synthesis of Small Molecule-Peptide Conjugate [21,22]

Peptide (1.0 equiv) and Posa(yne) (1.2 equiv) dissolved in 1 mL ultrapure water and 1 mL tert-butanol, mixed in a 5 mL centrifuge tube. Added catalytic amounts CuSO_4_·5H_2_O (1.0 equiv) and sodium L-ascorbate (5.0 equiv), followed by thorough vortexing and mixing. Ultrasonic reaction conducted at 37 °C for 90 min. Reaction progress monitored via HPLC. Upon completion, separation and purification executed via preparative high-performance liquid chromatography column (Shimadzu preparative HPLC system, Shimadzu Corporation, Kyoto, Japan) using: mobile phase A, 70% CH_3_CN/30% H_2_O/1‰ TFA solution; mobile phase B, 100% H_2_O/1‰ TFA solution; flow rate 10 mL·min^−1^; detection wavelengths 210 nm, 254 nm. Post-HPLC detection, fraction exhibiting purity > 95% was lyophilized, yielding pure product. Molecular weight resultant small molecule-peptide affix confirmed via MALDI-TOF-MS (Autoflex III, Bruker Daltonics, Billerica, MA, USA).

#### 3.3.1. Neutralisation Test of SARS-CoV-2 Omicron-Type XDV Pseudoviruses [23]

The SARS-CoV-2 (Omicron XDV strain) S was cloned into pNL4−3.Luc.R-E-vector, followed by pseudovirus collection via 293T/ACE2 target cells co-transfection using Lipofectamine 3000 (TCID_50_ = 1 × 10^6^/mL). The peptide (0.01–10 μM) was co-incubated with pseudovirus (MOI = 0.1) for 1 h to facilitate 293T/ACE2 target cells infection; luciferase activity was assessed after 48 h using Promega Luciferase Assay System, enabling IC_50_ calculation via GraphPad Prism 10 fitting. The cells were obtained from the Inner Mongolia Autonomous Region New Drug Screening Engineering Research Center in Inner Mongolia Medical University.

#### 3.3.2. SARS-CoV-2 S Protein-Mediated Cell–Cell Fusion Assay

Briefly, 1.5 × 10^4^ 293T effector cells were seeded per well of a 96-well plate, while 293T/ACE2 target cells were seeded at 1.5 × 10^5^ cells/mL in a 10 cm dish; all were incubated at 37 °C. The next day, effector cells were cotransfected with SARS-CoV-2 S protein-encoding and Dual-Split-Protein_1–7_ (DSP_1–7_) plasmids, and target cells with DSP_8–11_ plasmid, followed by 24 h of 37 °C incubation. Serially diluted compounds (appropriate ratio) were added to effector cells, incubated for 1 h. Target cells were resuspended to 3 × 10^5^ cells/mL in prewarmed medium with 17 ng/mL EnduRen (Promega, Madison, WI, USA), then incubated 30 min. 3 × 10^4^ target cells were transferred to effector cell wells; the mixture was centrifuged to enhance cell contact, then incubated 2 h. Luciferase activity was quantified, and IC_50_ values calculated as described previously. The cells were obtained from the Inner Mongolia Autonomous Region New Drug Screening Engineering Research Center in Inner Mongolia Medical University.

#### 3.3.3. Cytotoxicity Assays [24]

Briefly, 100 μL of 293T cell suspension (1 × 10^5^ cells/mL) was aliquoted into each well of a 96-well culture plate, and incubated at 37 °C in a 5% CO_2_ atmosphere for 12 h. Next, 5 μL of serially diluted peptide samples was added to each test well. Meanwhile, a blank control group (no peptide) and a positive control group (containing 5 μL of 10% Triton X-100) were established, and all groups were cultured at 37 °C with 5% CO_2_ for 48 h. Then, 10 μL of Cell Counting Kit-8 (CCK-8) solution (Yeasen, Shanghai, China) was added to each well. The CCK-8 assay, which utilizes the water-soluble tetrazolium salt WST-8, was performed according to the established colorimetric cell viability method. The cells were obtained from the Inner Mongolia Autonomous Region New Drug Screening Engineering Research Center in Inner Mongolia Medical University.

### 3.4. Method for Circular Dichroism (CD) [12]

Spectral scanning was performed using a JASCO J-1500 Circular Dichroism (CD) system. The quartz cuvette optical path length was 1 mm, and the scanning wavelength range was 190–260 nm at 25 °C, with a scanning speed of 50 nm/min, a response time of 1 s, a bandwidth of 1 nm, and an accumulation of 3 scans. All peptides and conjugates were prepared as 200 μM stock solutions in PBS (10 mM, pH 7.4). For binding analysis, HR2P and Posa-R8 were mixed at a 1:1 molar ratio (final concentration of 10 μM each, total volume 200 μL) and incubated at 37 °C for 60 min prior to measurement. For 6-HB disruption assays, HR1P(Omi) and HR2P were first mixed at a 1:1 molar ratio (10 μM each) in PBS, followed by the addition of the conjugate at various molar ratios (0.25:1, 0.5:1, 1:1, and 2:1, conjugate to HR2P), with a final total volume of 200 μL; mixtures were incubated at 37 °C for 60 min before spectral acquisition. The theoretical additive curves were calculated by summing the individual spectra of the separate components. Data analyses were performed using Origin 2022 Software.

### 3.5. Method for Native-Polyacrylamide Gel Electrophoresis (N-PAGE) [2]

The experiment used HR2P (derived from the SARS-CoV-2 HR2 region) as the target and HR1P(Omi) as the control. All peptides and conjugates were prepared as 600 μM stock solutions in PBS (10 mM, pH 7.4). For the 6-HB formation assay (positive control), HR1P(Omi) and HR2P were mixed at a 1:1 molar ratio (final concentration 100 μM each) in a total volume of 50 μL and incubated at 37 °C for 60 min. For the inhibition assays, the conjugates were added to the HR1P(Omi)/HR2P mixture at final molar ratios of 1:1, 4:1, and 8:1, with the total volume adjusted to 50 μL with PBS, and incubated at 37 °C for 60 min. An equal volume (50 μL) of 2× native loading buffer (125 mM Tris-HCl, pH 6.8, 20% glycerol, 0.02% bromophenol blue) was added to each sample, and 20 μL of each mixture was loaded per well onto a 12% native polyacrylamide gel (gel composition: 12% acrylamide/bis-acrylamide (29:1), 375 mM Tris-HCl, pH 8.8). Electrophoresis was performed at 90 V for 30 min, followed by 150 V for 2–3 h at room temperature, using Tris-glycine running buffer (25 mM Tris, 192 mM glycine, pH 8.3). Following electrophoresis, the gel was stained with Coomassie Brilliant Blue G-250 (Bio-Rad, Shanghai, China) for 30 min and destained with 10% acetic acid/40% methanol solution. The gel was then imaged using a gel documentation system (Sage Creation Ltd., Beijing, China).

### 3.6. Method for Size Exclusion Chromatography (SE-HPLC) [12]

For the 6-HB formation control, HR1P(Omi) and HR2P were mixed at a 1:1 molar ratio to achieve a final concentration of 25 μM each (total 50 μM complex) in PBS (10 mM, pH 7.4). For inhibition assays, the conjugates were added to the HR1P(Omi)/HR2P mixture at final molar ratios of 1:1, 4:1, and 8:1. All samples (total volume 100 μL) were incubated at 37 °C for 60 min prior to injection. Analysis used a Phenomenex BioSep-SEC-S2000 column (300 mm × 7.8 mm, 5 μm) under these conditions: Mobile phase: PBS (10 mM, pH 7.4); Flow rate: 0.8 mL/min; Detection wavelength: 210 nm; Injection volume: 20 μL; Elution mode: isocratic; Sample analysis time: 30 min; Column equilibration time: 30 min per injection. All samples were filtered through a 0.22 μm membrane prior to injection.

### 3.7. Method for Molecular Docking [25]

AutoDock Vina 1.2.2 facilitated the exploration of interactions between conjugates and the target. Molecular structures of conjugates were drawn using ChemDraw 21.0 and subsequently minimized in Chem3D. The 6-HB crystal structure (SARS-CoV-2, PDB ID: 6LXT) was obtained from the Protein Date Bank and energy-minimized using MOE 2024.06 docking software. For molecular docking, all protein and molecular files were converted to PDBQT format, water molecules were removed, and polar hydrogens were added. Grid boxes centered on protein structural domains were employed to allow free molecular motion, with the docking pocket defined as a 30 Å × 30 Å × 30 Å cube featuring a 0.05 nm grid spacing. Docking conformations were analyzed and visualized using PyMOL v3.0 software.

### 3.8. Method for Isothermal Titration Calorimetry (ITC) [26]

ITC was performed to detect the conjugate and lipid-binding activity. To detect the conjugates and lipid-binding activity, the large unilamellar vesicles (LUVs) of Lecithin was used. Briefly, Lecithin (150 μM), degassed under vacuum prior to use, were injected into the compound solution (15 μM). The experiments were performed using a MicroCal ITC200 system (GE, Alpharetta, GA, USA) fortitration, with the following experimental parameters: total injection, 20 drops; drop volume, 2 μL; syringe concentration, 150 μM; compound concentration, 15 μM; sample pool temperature, 25 °C; energy reference, 3 μCal/s; titration delay, 60 s; stirring speed, 750 rpm; drop volume, 2 μL; titration time, 4 s; two-drop interval, 120 s; data collection interval, 5 s. Data acquisition and analyses were performed using MicroCal PEAQ-ITC Analysis Software v1.50.

### 3.9. Phase I Metabolic Stability Assay [27]

The assay was performed following the instructions of the Phase I Metabolic Stability Research Kit (IPHASE, Suzhou, China). The incubation conditions and data analysis followed the established liver microsomal stability protocol described previously. Solution A from the kit was pre-incubated at 37 °C for 5 min. Separately, Solution B was prepared by combining PBS (100 mM), the test compound (or a positive control substrate), and liver microsomes. The above two solutions were then mixed and the reaction was initiated by immediate transfer to a 37 °C water bath. At predetermined time intervals (0, 5, 10, 15, 30, 60 min), aliquots of the incubation mixture were withdrawn and quenched with an equal volume of stop solution. All samples were prepared in triplicate. The remaining amount of substrate at each time point was quantified by RP-HPLC (Shimadzu preparative HPLC system, Shimadzu Corporation, Kyoto, Japan). The percentage of substrate remaining was calculated, and the elimination rate constant (k) was determined via linear regression analysis. This value was subsequently used to calculate the Elimination half-life and Hepatic microsomal intrinsic clearance.

## 4. Results and Discussion

### 4.1. Conjugates Block Pseudotyped SARS-CoV-2 Omicron XDV Infection

**Figure 2:**
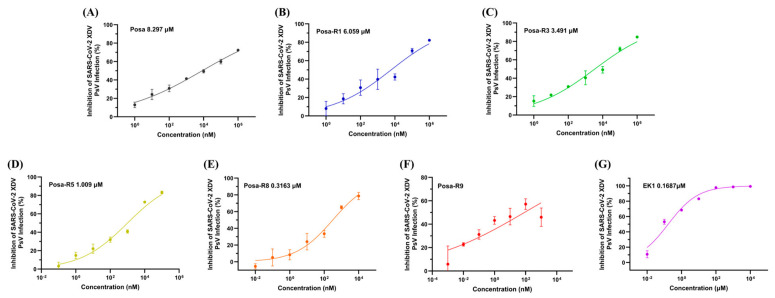
Inhibitory activity of compounds in pseudovirus infection assays against SARS-CoV-2 Omicron XDV strain. (**A**) Posa; (**B**) Posa-R1; (**C**) Posa-R3; (**D**) Posa-R5; (**E**) Posa-R8; (**F**) Posa-R9; (**G**) EK1 (the IC_50_ value for Posa-R9 is not reported because the compound exhibited reduced inhibitory activity at higher concentrations, indicative of cytotoxicity associated with excessive arginine residues, which resulted in a non-standard dose–response curve that precluded reliable IC_50_ fitting).

We evaluated the antiviral activity of the conjugates using a pseudotyped SARS-CoV-2 Omicron XDV strain infection assay. Pseudovirus assays effectively model viral entry and are widely used to assess inhibitor potency [23]. As shown in Figure 2, Posa-R1 exhibited anti-SARS-CoV-2 activity comparable to that of Posa, while the activity of other conjugates increased with polyarginine chain length. However, Posa-R9 showed reduced inhibitory activity at higher concentrations, suggesting potential cytotoxicity associated with excessive arginine residues. Posa-R8 demonstrated potent activity (IC_50_ = 0.3163 μM) comparable to that of the positive control EK1, with no significant cytotoxicity (Supplementary Figure S29). Therefore, we selected Posa-R8 to investigate anti-SARS-CoV-2 mechanism.

### 4.2. Conjugates Interact with the SARS-CoV-2 S HR2 Region and Disrupt 6-HB Formation

**Figure 3:**
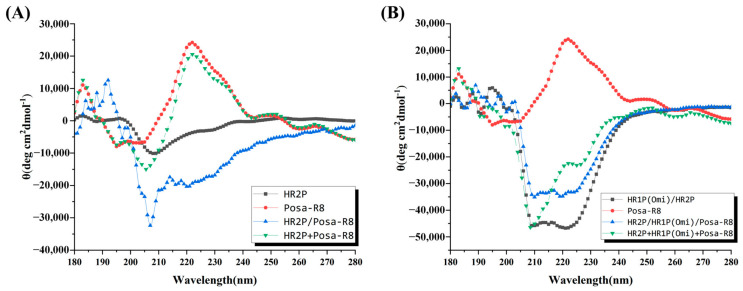
(**A**) CD spectrum of Posa-R8 and HR2P complex (HR2P/Posa-R8 represents the Posa-R8 and HR2P mixed solution); (**B**) CD spectrum of Posa-R8 and HR1P(Omi)/HR2P complex (HR2P/HR1P(Omi)/Posa-R8 represents the Posa-R8, HR1P(Omi) and HR2P mixed solution).

**Figure 4:**
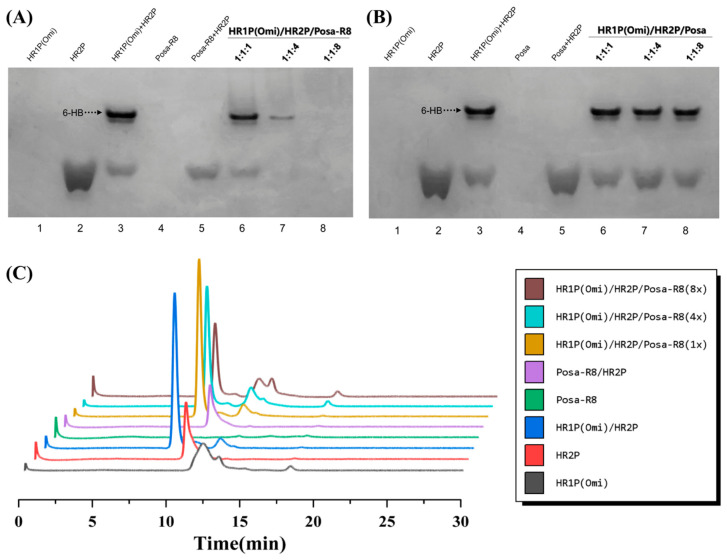
(**A**) N-PAGE analysis of Posa-R8 with target peptide (HR1P(Omi)/HR2P/Posa-R8 represents the Posa-R8, HR1P(Omi) and HR2P mixed solution); (**B**) N-PAGE analysis of Posa with target peptide (HR1P(Omi)/HR2P/Posa represents the Posa, HR1P(Omi) and HR2P mixed solution); (**C**) SE-HPLC analysis of Posa-R8 with target peptide (HR1P(Omi)/HR2P/Posa-R8 represents the Posa-R8, HR1P(Omi) and HR2P mixed solution).

Circular dichroism (CD) spectroscopy was used to assess interactions between Posa-R8 and the HR2 region. HR2P (derived from the HR2 region of the Omicron variant) and HR1P(Omi) (derived from the HR1 region of the Omicron variant) were used as target and control peptides, respectively. As shown in Figure 3A, Posa-R8 alone exhibited no helical structure, while HR2P alone showed low helicity. However, when Posa-R8 was co-incubated with HR2P (which is present as a monomer in this assay, not assembled into the 6-HB), the experimental blue curve exhibited significantly higher helicity than the green theoretical additive curve. This increased helicity indicates that Posa-R8 binds directly to the HR2 monomer and induces an α-helical conformation upon complexation, confirming formation of a Posa-R8/HR2P complex. It is important to distinguish this binding assay from the 6-HB disruption assay: in Figure 3A, we measure induced helicity upon binding to free HR2; whereas Figure 3B shows the loss of helicity following the disruption of the pre-assembled 6-HB. In Figure 3B, the 6-HB complex formed by the mixture of HR1P(Omi) and HR2P exhibits a standard helical curve (black curve), whereas the addition of Posa-R8 results in a significant reduction in the 6-HB helix content (blue curve), which deviates considerably from the green theoretical curve, indicating that Posa-R8 disrupts the 6-HB assembly formed by HR1P(Omi) and HR2P. Similar results were obtained for other conjugates (Supplementary Figures S15–S18). Native polyacrylamide gel electrophoresis (N-PAGE) and size-exclusion high-performance liquid chromatography (SE-HPLC) were employed to further validate aforementioned CD experiment findings. In N-PAGE results (Figure 4A), HR2P appeared as a distinct band (lane 2), while HR1P(Omi) and Posa-R8 showed no bands due to positive charge (lanes 1 and 4). Co-incubation of HR2P and HR1P(Omi) formed a stable 6-HB complex (lane 3). The administration of Posa-R8 to the HR2P solution reduced the HR2P band intensity (lane 5), while gradually increasing the Posa-R8 concentration in a mixture of HR2P and HR1P(Omi) caused the 6-HB band to progressively diminish in a concentration-dependent manner (lanes 6–8). At the highest molar ratio tested (lane 8), the 6-HB band was reduced to less than 5% of the control intensity (lane 3), becoming virtually undetectable under the staining conditions. These results indicate that Posa-R8 binds to HR2P and significantly destabilizes 6-HB. In contrast, the Posa showed almost no tendency to disrupt 6-HB but exhibited weak binding to HR2P (Figure 4B). In the SE-HPLC, compounds show different retention times correlating with molecular volume, larger volumes mean shorter retention times. As shown in Figure 4C, the HR2P eluted at ~10.5 min (red curve), while 6-HB complex eluted earlier at ~9.2 min (blue curve). Aligned with N-PAGE, Posa-R8 reduces the HR2P peak and gradually weakens the 6-HB peak in a concentration-dependent manner. All of the above experimental systems collectively confirm that Posa-R8 can bind to the HR2 region of S and disrupt the endogenous 6-HB formation. Similar results were obtained for other conjugates (Supplementary Figures S19–S26).

### 4.3. Molecular Docking of Conjugates with the 6-HB Target

Molecular docking using AutoDock Vina was performed to investigate the binding modes of the conjugates with the 6-HB target (PDB ID: 6LXT). First, the binding of Posa to the 6-HB was validated using Itraconazole and Voriconazole as controls. As shown in Supplementary Figure S27, Posa can bind to the 6-HB core region, forming key hydrogen bonds with Asp1184 and Glu1182 and engaging in hydrophobic interactions with Lys933, Gln935, Asn1187, and Glu1188. In contrast, Itraconazole and Voriconazole cannot bind to the target core, only recognize the 6-HB terminal end. Therefore, the molecular docking model of Posa reliably confirms the specificity of Posa binding to the 6-HB helical core region.

**Figure 5:**
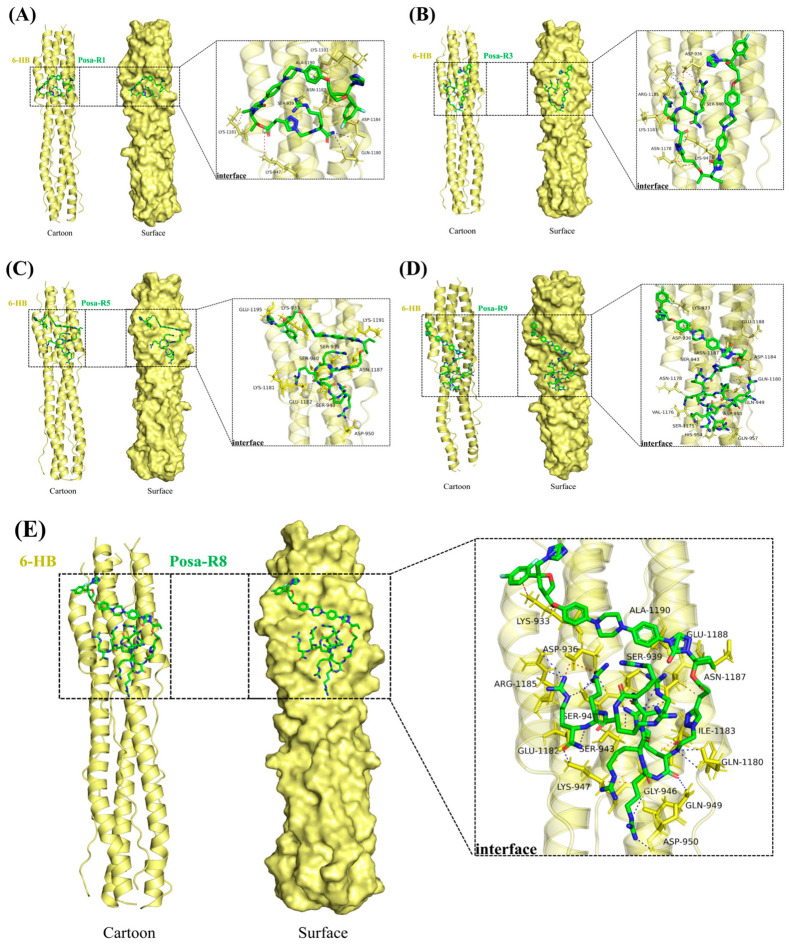
(**A**) Molecular docking analyses of Posa-R1 with the 6-HB region. (**B**) Molecular docking analyses of Posa-R3 with the 6-HB region. (**C**) Molecular docking analyses of Posa-R5 with the 6-HB region. (**D**) Molecular docking analyses of Posa-R9 with the 6-HB region. (**E**) Molecular docking analyses of Posa-R8 with the 6-HB region (important residues are shown as sticks and labeled; the blue dashed lines are the hydrogen bonds, red dashed lines are the salt bridges, magenta dashed lines are the hydrophobic interactions, and orange dashed lines are the cation–π interaction).

Subsequently, we proceeded to utilise this molecular docking to conduct a detailed investigation into the interactions between ligand conjugates and target 6-HB. The results revealed that all conjugates exhibited multiple interactions with the 6-HB residue sites (Figure 5). Posa-R1 (Figure 5A): Arginine moiety forms hydrogen bonds with Gln1180, Asp1184, Asn1187 and Ser939 residues in the 6-HB region. The posaconazole hydrophilic moiety forms a salt bridge with Lys947 residue, while its hydrophobic moiety exhibits extensive hydrophobic interactions with side chains of Lys1181, Asp1184, Asn1187 and Ala1190 in the 6-HB. Posa-R3 (Figure 5B): Its polyarginine moiety forms hydrogen bonds with Arg1185 and Ser940 residues and a salt bridge with Asp936 residue in the 6-HB region, while its posaconazole moiety forms hydrogen bonds with Lys947 and Asn1178 residues in the same region. Additionally, the remaining hydrophobic moiety exhibits hydrophobic interactions with Asn1178 and Lys1181 residues in the 6-HB region. Posa-R5 (Figure 5C): Its polyarginine moiety forms hydrogen bonds with Ser939, Ser940, Ser943, Lys1181, Glu1182 and Glu1195 residues in the 6-HB region, and forms salt bridges with Asp950, Glu1182 and Glu1195 residues in the same region. The remaining hydrophobic moiety exhibits hydrophobic interactions with Lys933, Asn1187 and Lys1191 residues in the 6-HB region. Posa-R8 (Figure 5E): The polyarginine moiety forms a range of hydrogen bonds with Ser939, Ser940, Ser943, Gly946, Lys947, Gln949, Asp950, Gln1180, Glu1182, Arg1185 and Asn1187 residues in the 6-HB region, and forms a salt bridge with Asp936 residue in the same region. Its posaconazole moiety forms a cation-π interaction with Lys933 in the 6-HB region, and its hydrophobic moiety exhibits hydrophobic interactions with side chains of other residues in the 6-HB region, such as Lys933, Lys947 and Ile1183. Posa-R9 showed similar interactions to Posa-R8 (Figure 5D). These results indicate that polyarginine groups synergistically enhance the affinity of the conjugate for the 6-HB target, thereby promoting antiviral activity.

### 4.4. Posa-R8 Exhibits Improved Phase I Metabolic Stability Compared to EK1

Hepatic microsomes are rich in phase I metabolic enzymes, among which the microsomal mixed-function oxidase system—centered on cytochrome P450 (CYP450) isoforms as its catalytic core—acting as the pivotal machinery for xenobiotic biotransformation. To mimic this in vitro phase I metabolic process, we established a functional assay system by adding the essential cofactor NADPH [28], allowing us to assess phase I metabolic stability of Posa-R8 via in vitro incubation, with EK1 as the positive control. Table 1 summarizes the elimination half-life (Ehl) and hepatic microsomal intrinsic clearance (Hmic) of two compounds: Posa-R8 exhibited a notably prolonged Ehl of 52.11 min, whereas EK1 displayed a shorter Ehl of 31.22 min. Correspondingly, Posa-R8 showed a lower Hmic value (26.60 μL·min^−1^·mg^−1^) relative to EK1 (44.39 μL·min^−1^·mg^−1^). Our data reveal that, following polyarginine modification, such Posa-R8 exhibit more favorable phase I metabolic properties relative to the positive control EK1, underscoring the value of this modification strategy for optimizing Posa pharmacokinetics.

### 4.5. Discussion

**Figure 6:**
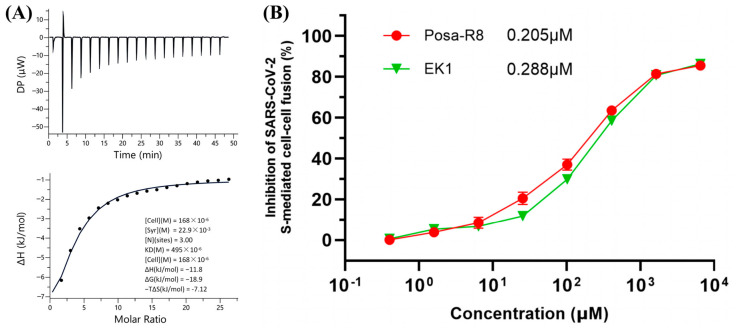
(**A**) Analysis of the binding of Posa-R8 to a lipid bilayer. (**B**) Inhibition of SARS-CoV-2 S-mediated cell–cell fusion of Posa-R8 and EK1.

Existing research has shown that SARS-CoV-2 can enter host cells either directly at the plasma membrane or via endocytosiss [29,30]. Unfortunately, our platform lacks the capabilities to evaluate a model of SARS-CoV-2 entry via endocytosis. Therefore, following the previous literature [26,31], we employed Isothermal titration calorimetry (ITC) experiment and the SARS-CoV-2 S-protein-mediated cell–cell fusion model to assess whether the conjugate is capable of accumulating in the membrane fusion high-lipid environment. ITC was used to assess the lipid bilayer binding affinity of Posa-R8 using POPC large unilamellar vesicles (LUVs). Figure 6A shows that when POPC LUVs are injected into Posa-R8 solution, the binding affinity constant reached 4.95 × 10^−4^ M. Negative ΔH and ΔG values indicated an exothermic, spontaneous binding process. In contrast, Posa alone showed no significant thermal signal (Supplementary Figure S28), confirming the critical role of the polyarginine moiety in membrane binding. Furthermore, Posa-R8 effectively inhibited SARS-CoV-2 S-mediated cell–cell fusion, with activity comparable to EK1 (Figure 6B). Overall, these results show that Posa-R8 exhibits strong lipid bilayer binding—in contrast to posaconazole alone, which showed no significant binding—and, combined with its potent inhibition of cell fusion, provide strong evidence that polyarginine conjugation enhances membrane interactions.

## 5. Conclusions

This study successfully developed novel SARS-CoV-2 fusion inhibitors by conjugating posaconazole with polyarginine to overcome its poor membrane-binding affinity and modest target affinity. Among the conjugates, Posa-R8 exhibited potent antiviral activity against the Omicron XDV pseudovirus (IC_50_ = 0.3163 μM), comparable to the positive inhibitor EK1, with no apparent cytotoxicity. Mechanistic studies (CD, N-PAGE, SE-HPLC, and molecular docking) confirmed that Posa-R8 disrupts 6-HB formation through multiple non-covalent interactions with the HR2 region. Furthermore, Posa-R8 showed superior phase I metabolic stability (t_1/2_ = 52.11 min) compared to EK1 (t_1/2_ = 31.22 min), along with strong lipid-bilayer affinity attributed to the polyarginine moiety. Nevertheless, this study has limitations: the antiviral activity was assessed only in pseudovirus-based assays and against a single Omicron sublineage (XDV), and no in vivo efficacy data are yet available. Additionally, our current platform does not address the endocytic entry pathway. Future work will focus on evaluating Posa-R8 in authentic SARS-CoV-2 infection models, testing its activity against a broader panel of variants, and performing in vivo pharmacokinetic and efficacy studies in animal models.

## Figures and Tables

**Table 1:** Elimination half-life and Hepatic microsomal intrinsic clearance for Posa-R8 and EK1 ^a^.

Compound	Ehl (min) ^b^	Hmic (μL·min^−1^·mg^−1^) ^c^
Posa-R8	52.11	26.60
EK1	31.22	44.39

^a^ The chromatographic peak area was used as the calculation data. Take the zero concentration of the test substance as 100%, and compare the concentration at each time point with the zero concentration to obtain the remaining percentage. The natural logarithm of the remaining percentage of substrate at each time point is linearly regressed with the incubation time to obtain the slope *K*. ^b^ Elimination half-life = 0.693/*K*. ^c^ Hepatic microsomal intrinsic clearance = [0.693/Elimination half-life] × [Incubation volume (mL)/Liver microsomal mass (mg)].

## Data Availability

The original contributions presented in this study are included in the article/Supplementary Materials. Further inquiries can be directed to the corresponding author.

## Supplementary Materials

The following supporting information can be downloaded at https://www.mdpi.com/article/10.3390/v18070737/s1, Figure S1: ^1^H NMR spectrogram of Posa(yne) (DMSO-*d*_6_); Figure S2: ^13^C NMR spectrogram of Posa(yne) (DMSO-*d*_6_); Figure S3: MALDI-TOF-MS spectrogram of Posa(yne); Figure S4: RP-HPLC diagram of Posa(yne); Figure S5: MALDI-TOF-MS spectrogram of Posa-R1; Figure S6: RP-HPLC diagram of Posa-R1; Figure S7: MALDI-TOF-MS spectrogram of Posa-R3; Figure S8: RP-HPLC diagram of Posa-R3; Figure S9: MALDI-TOF-MS spectrogram of Posa-R5; Figure S10: RP-HPLC diagram of Posa-R5; Figure S11: MALDI-TOF-MS spectrogram of Posa-R8; Figure S12: RP-HPLC diagram of Posa-R8; Figure S13: MALDI-TOF-MS spectrogram of Posa-R9; Figure S14: RP-HPLC diagram of Posa-R9; Figure S15: (A) CD spectrum of Posa-R1 and HR2P complex (B) CD spectrum of Posa-R1 and HR1P(Omi)/HR2P complex; Figure S16: (A) CD spectrum of Posa-R3 and HR2P complex (B) CD spectrum of Posa-R3 and HR1P(Omi)/HR2P complex; Figure S17: (A) CD spectrum of Posa-R5 and HR2P complex (B) CD spectrum of Posa-R5 and HR1P(Omi)/HR2P complex; Figure S18: (A) CD spectrum of Posa-R9 and HR2P complex (B) CD spectrum of Posa-R9 and HR1P(Omi)/HR2P complex; Figure S19: N-PAGE analysis of Posa-R1 with target peptide; Figure S20: N-PAGE analysis of Posa-R3 with target peptide; Figure S21: N-PAGE analysis of Posa-R5 with target peptide; Figure S22: N-PAGE analysis of Posa-R9 with target peptide; Figure S23: SE-HPLC analysis of Posa-R1 with target peptide; Figure S24: SE-HPLC analysis of Posa-R3 with target peptide; Figure S25: SE-HPLC analysis of Posa-R5 with target peptide; Figure S26: SE-HPLC analysis of Posa-R9 with target peptide; Figure S27: (A) Molecular docking analyses of Posa with the 6-HB region; (B) Molecular docking analyses of Itraconazole with the 6-HB region; (C) Molecular docking analyses of Voriconazole with the 6-HB region; Figure S28: Analysis of the binding of Posa to a lipid bilayer; Figure S29: Cytotoxicity diagram of Posa-R8 and EK1.
