# Supplementary material for: Discovery of Novel SARS-CoV-2 Fusion Inhibitors—Posaconazole-Polyarginine Conjugates
Source: Viruses. 2026 Jul 2;18(7):737. doi: 10.3390/v18070737 (PMC13431547; doi:10.3390/v18070737)
Supplement: Supplementary file 1 [file viruses-18-00737-s001.zip › viruses-4388926-supplementary.pdf]

Supporting Information

Discovery of Novel SARS-CoV-2 Fusion Inhibitors—Posaconazole-Polyarginine Conjugates

Yihui Jin[1,#], Lili Qu[3,#], Xin Gao[1], Xiao Qi[1], Dongmin Zhao[1], Lu Ga[1], Yan Zhao[1], Guodong Liang[1,2,4,*], Yunfeng Xiao[1,*], Yuheng Ma[1,*]

(1. Key Laboratory for Candidate Drug Design and Screening Based on Chemical Biology, College of Pharmacy, Inner Mongolia Medical University, Hohhot, P.R.China; 2. State Key Laboratory of Natural and Biomimetic Drugs, Peking University, Beijing, P.R.China; 3. Department of Pharmacy, The Affiliated Hospital of Inner Mongolia Medical University, Hohhot, P.R.China; 4. Peptide Drugs Research and Development Center, Zhen-Xiang Technology Co., Ltd, Hohhot, P.R.China)

* Corresponding author. Email: 20220821@immu.edu.cn to Guodong Liang, 20060209@immu.edu.cn to Yunfeng Xiao, and 20120311@immu.edu.cn to Yuheng Ma.

# These authors contributed equally to this work.

Content:

1. NMR spectrogram of compounds....................3
2. MALDI-TOF-MS spectrogram and RP-HPLC diagram of compounds....................4
3. CD spectrogram of compounds....................10
4. N-PAGE diagram of compounds....................12
5. SE-HPLC diagram of compounds....................14
6. Molecular docking diagram of compounds....................16
7. ITC diagram of compounds....................17
8. Cytotoxicity of compounds....................18

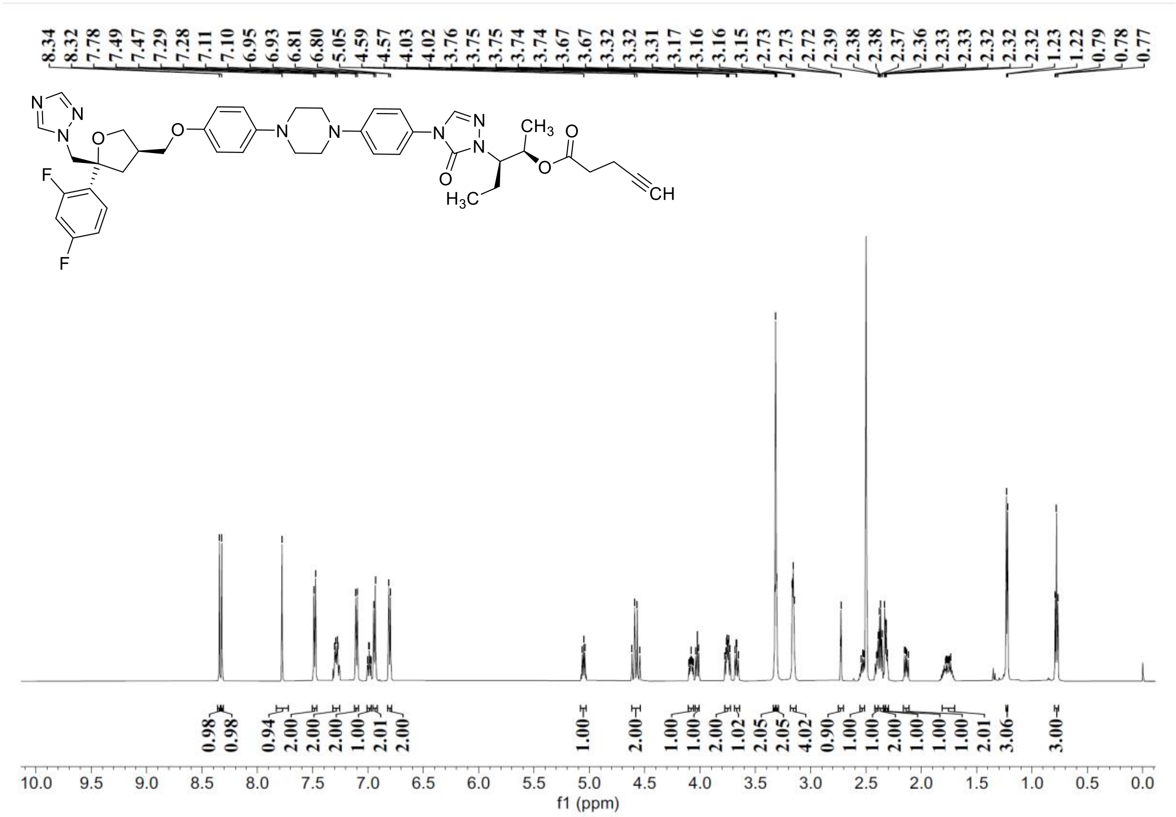

Figure S1. ^{1}H NMR spectrogram of Posa(yne) (DMSO-d_6)

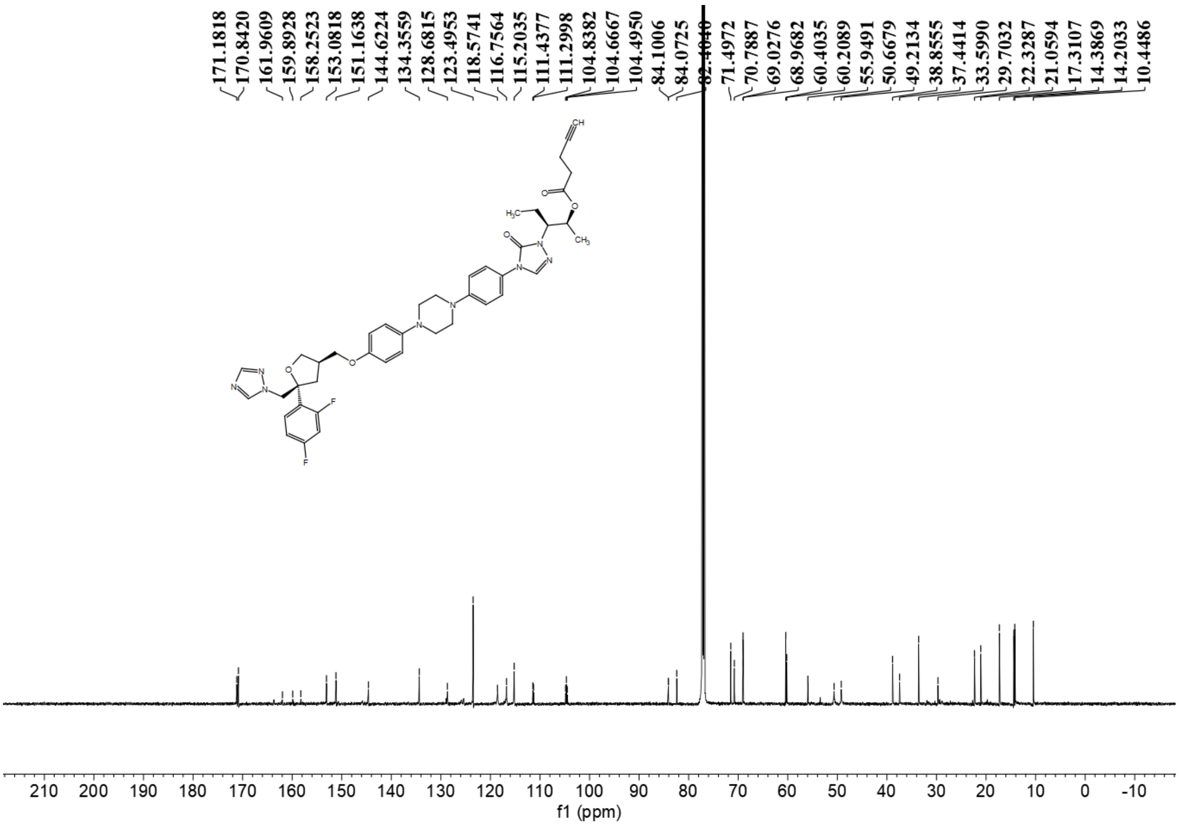

Figure S2. ^{13}C NMR spectrogram of Posa(yne) (DMSO-d_6)

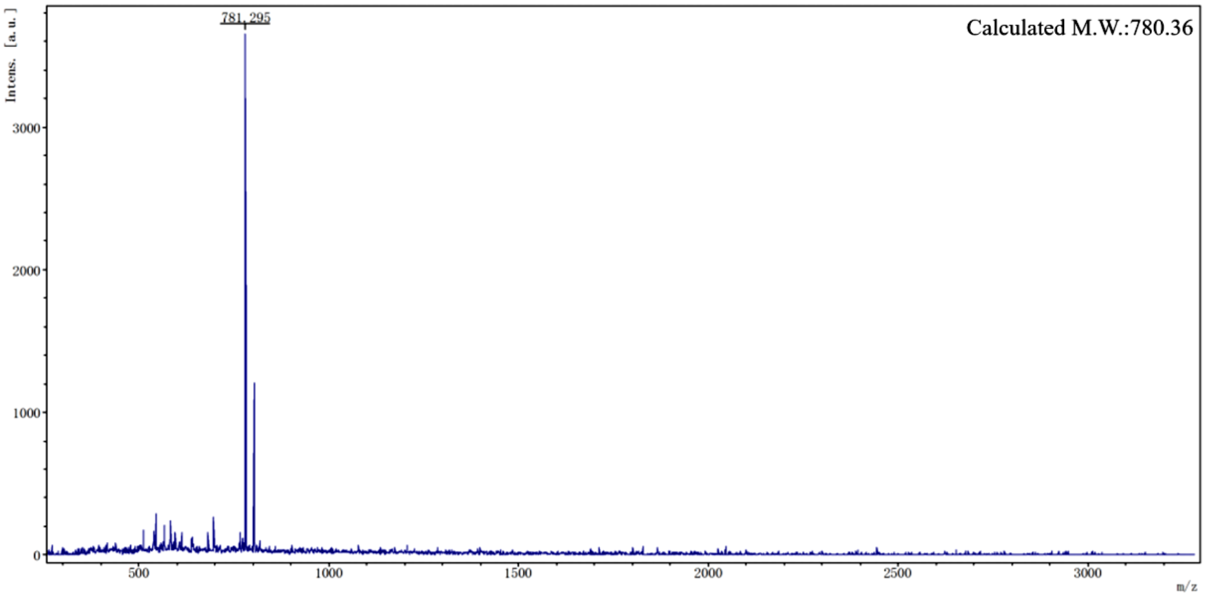

Figure S3. MALDI-TOF-MS spectrogram of Posa(yne)

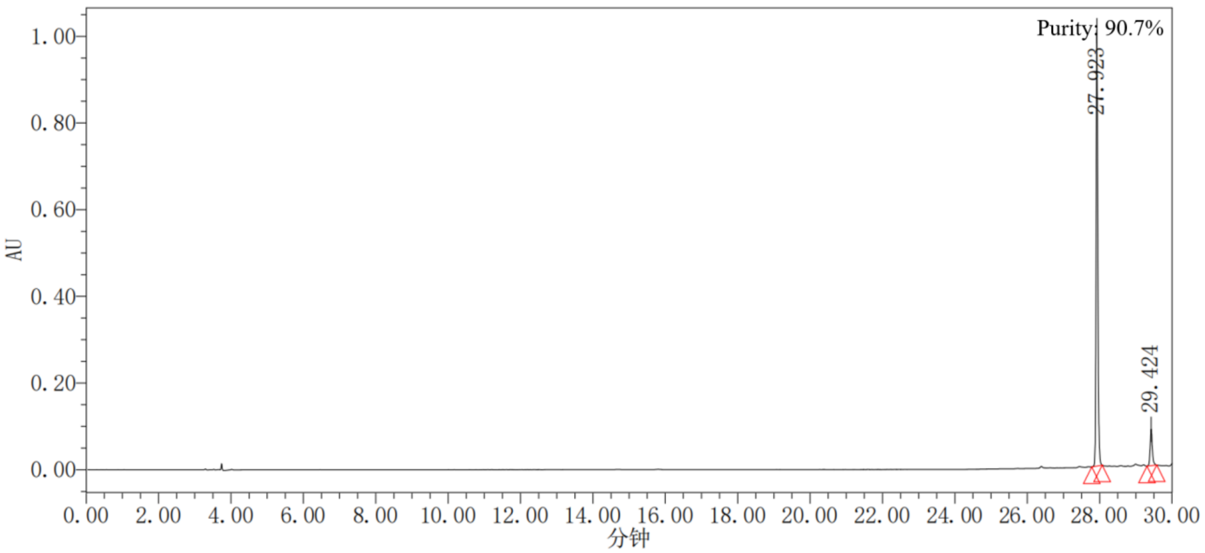

Figure S4. RP-HPLC diagram of Posa(yne)

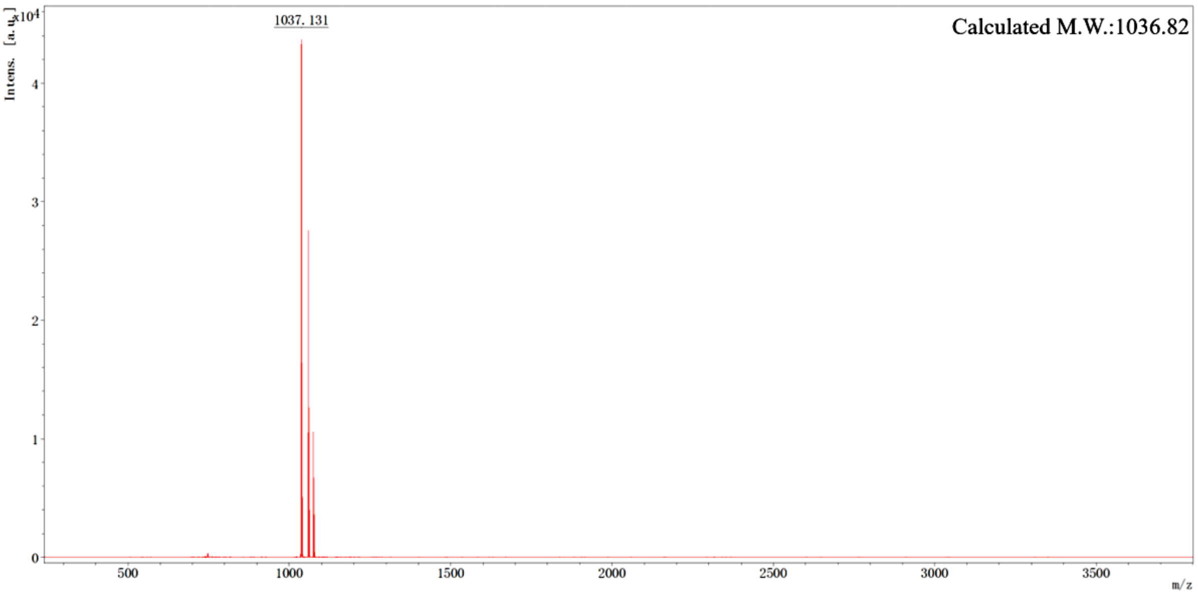

Figure S5. MALDI-TOF-MS spectrogram of Posa-R1

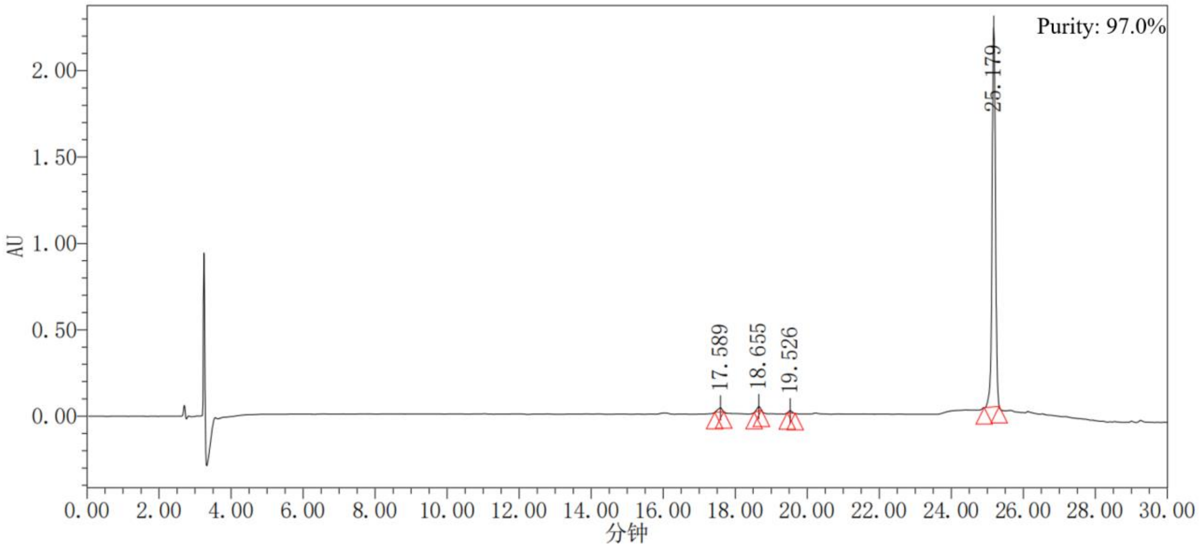

Figure S6. RP-HPLC diagram of Posa-R1

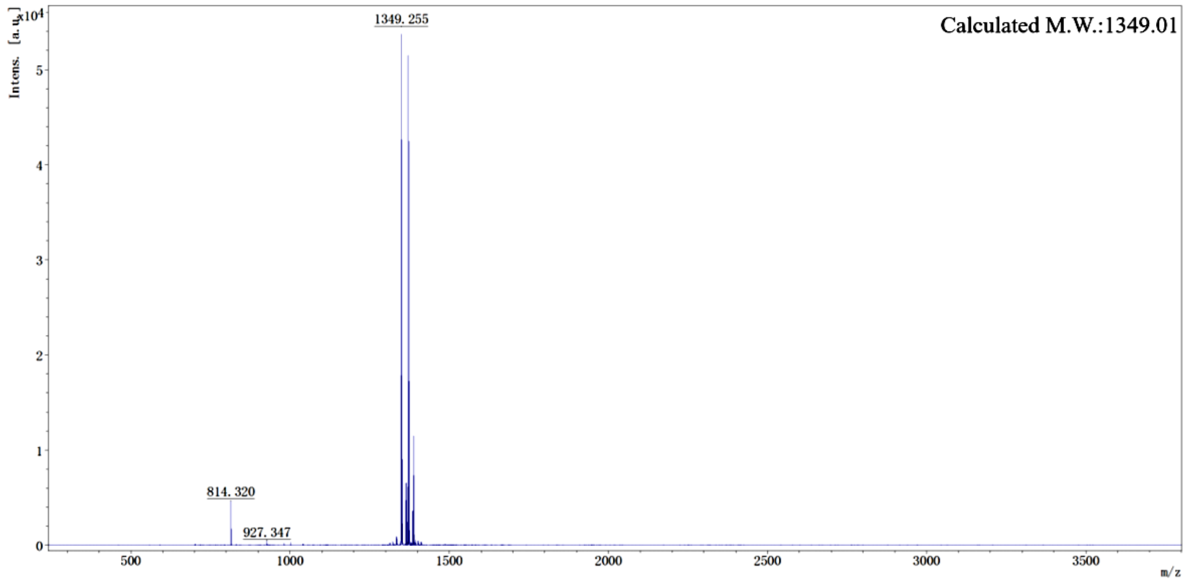

Figure S7. MALDI-TOF-MS spectrogram of Posa-R3

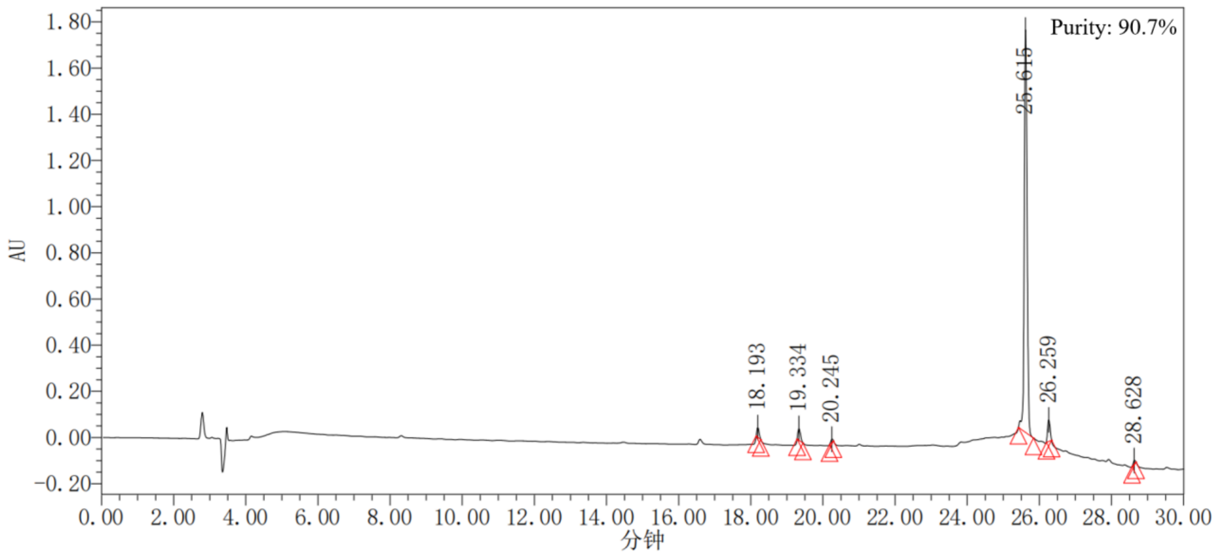

Figure S8. RP-HPLC diagram of Posa-R3

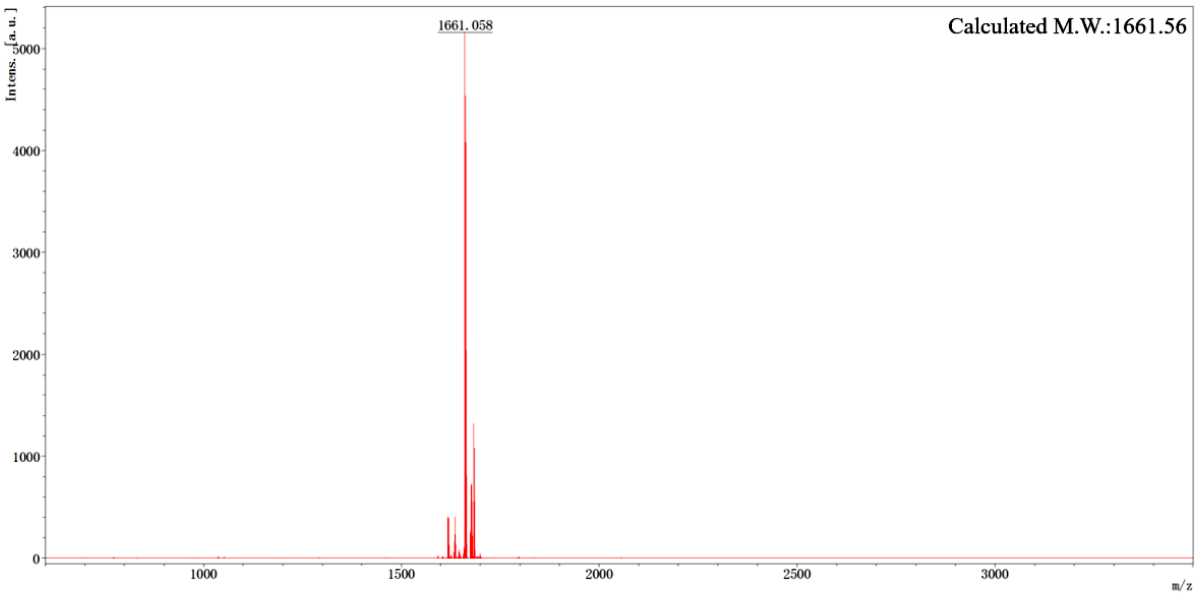

Figure S9. MALDI-TOF-MS spectrogram of Posa-R5

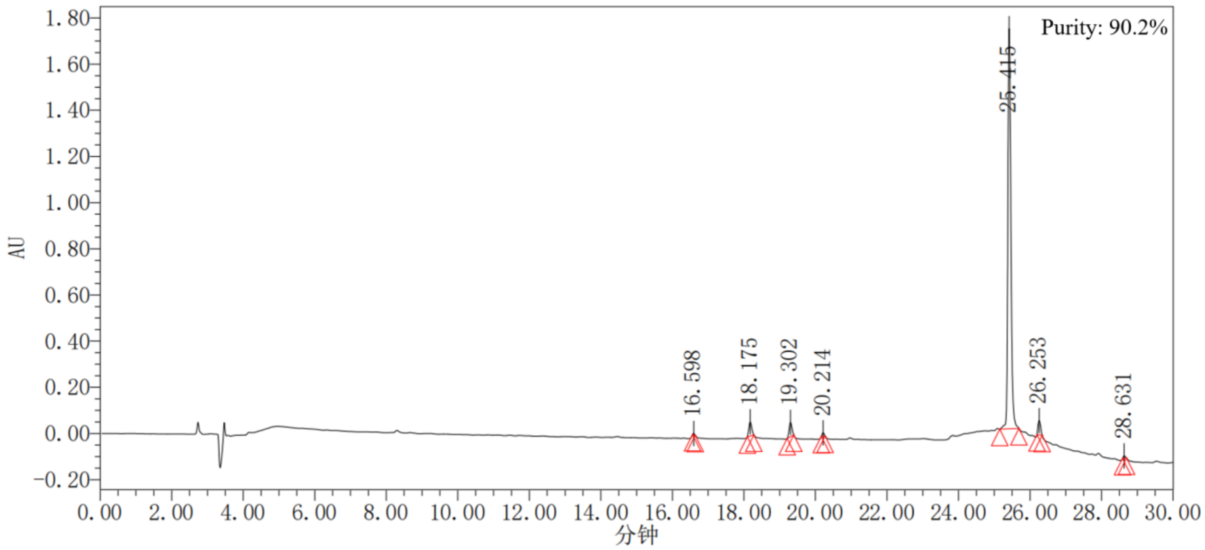

Figure S10. RP-HPLC diagram of Posa-R5

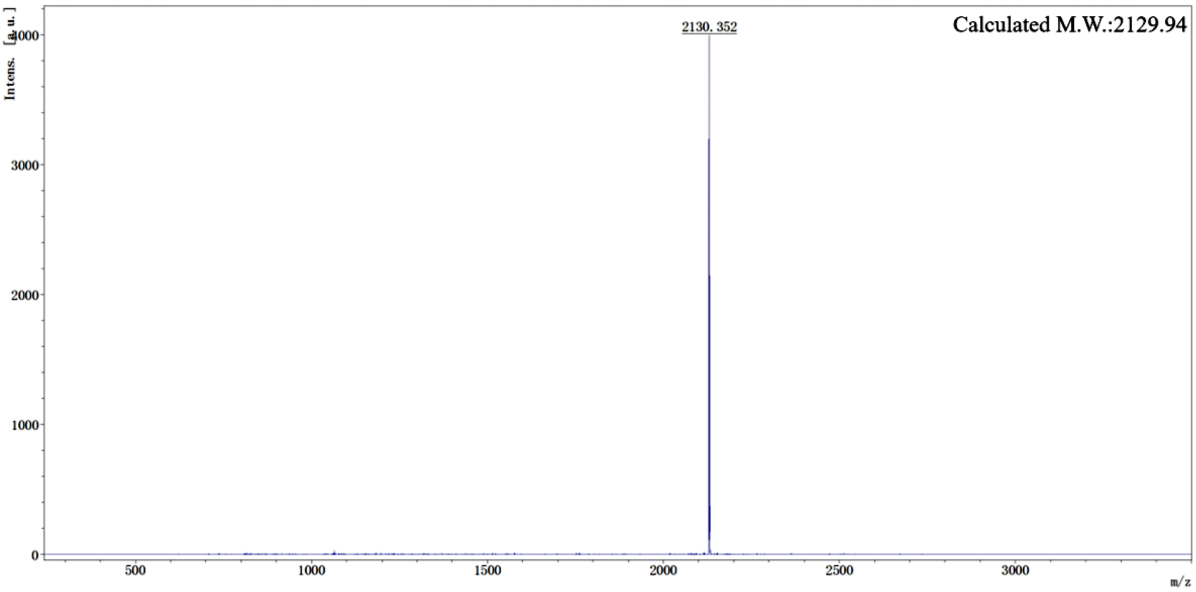

Figure S11. MALDI-TOF-MS spectrogram of Posa-R8

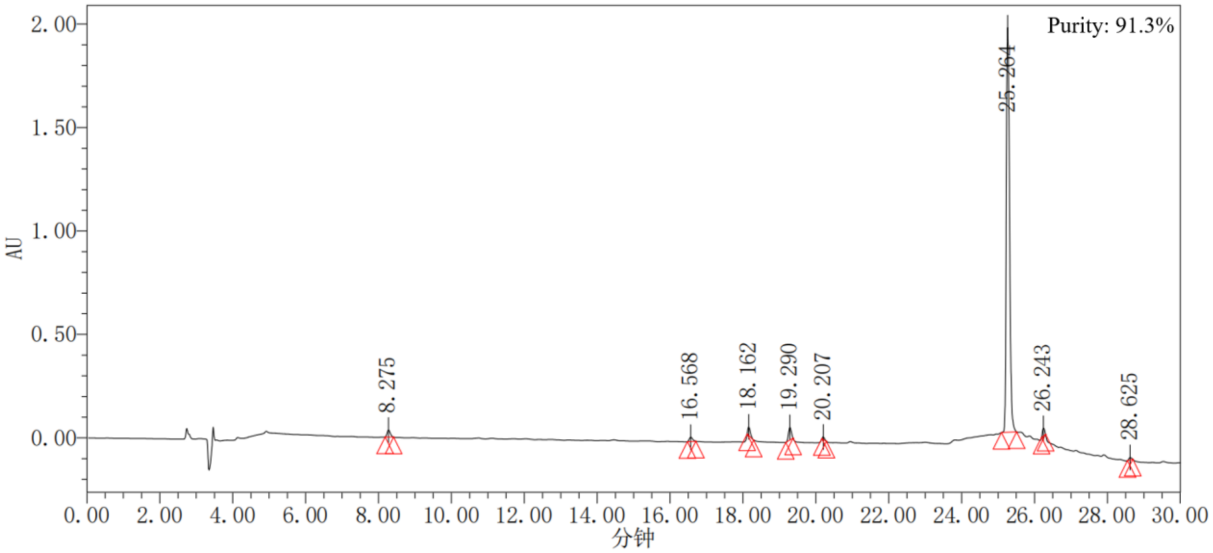

Figure S12. RP-HPLC diagram of Posa-R8

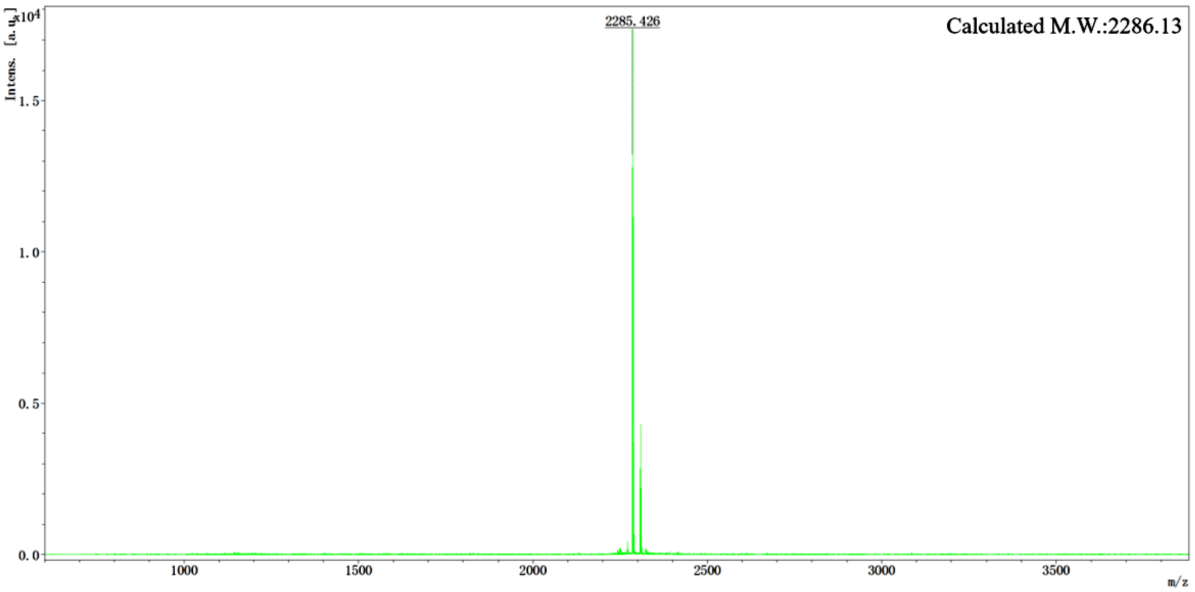

Figure S13. MALDI-TOF-MS spectrogram of Posa-R9

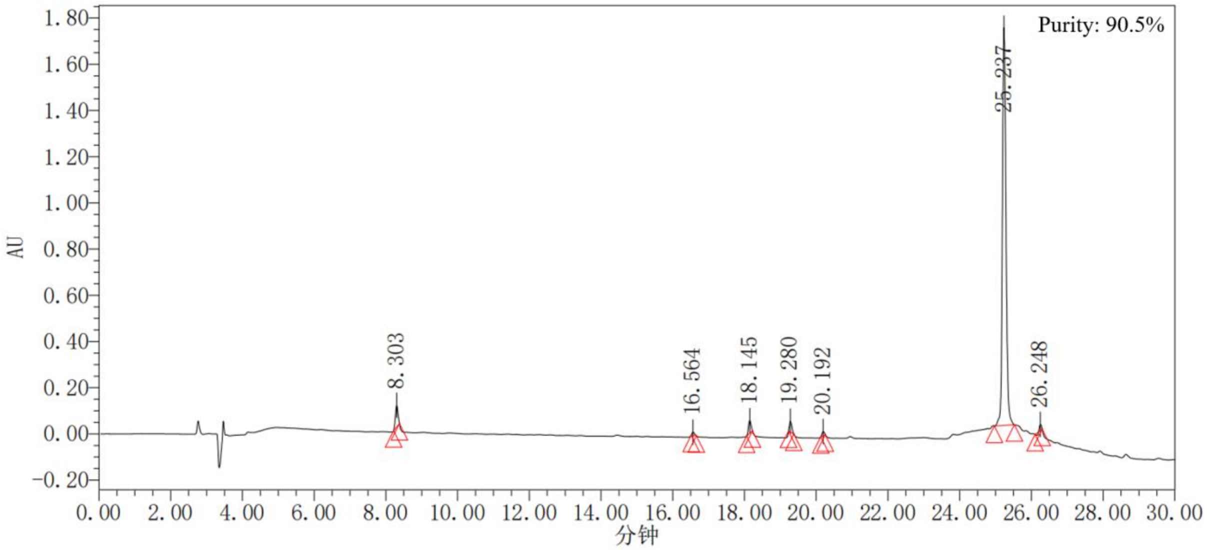

Figure S14. RP-HPLC diagram of Posa-R9

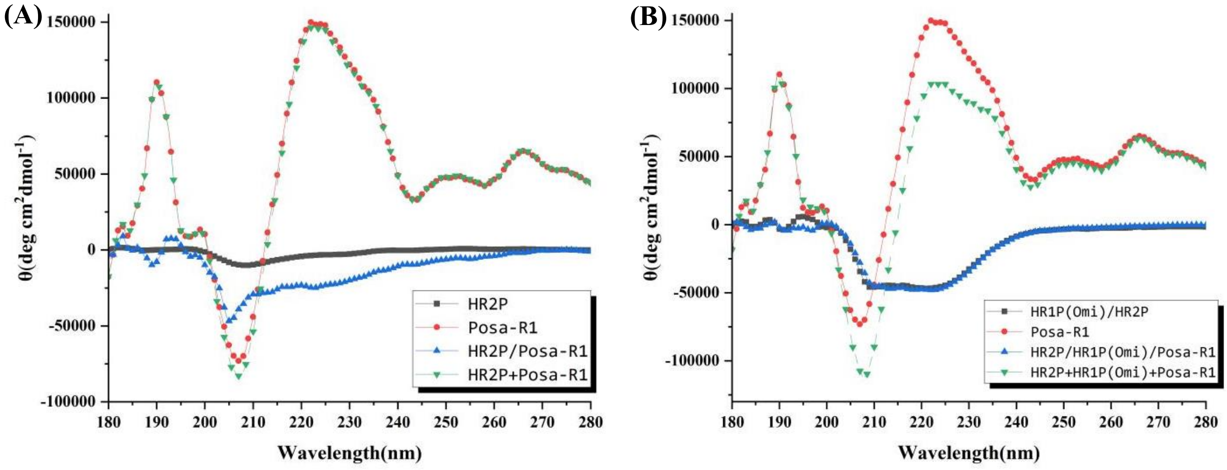

Figure S15. (A) CD spectrum of Posa-R1 and HR2P complex (HR2P/Posa-R1 represents the Posa-R1 and HR2P mixed solution); (B) CD spectrum of Posa-R1 and HR1P(Omi)/HR2P complex (HR2P/HR1P(Omi)/Posa-R1 represents the Posa-R1, HR1P(Omi) and HR2P mixed solution).

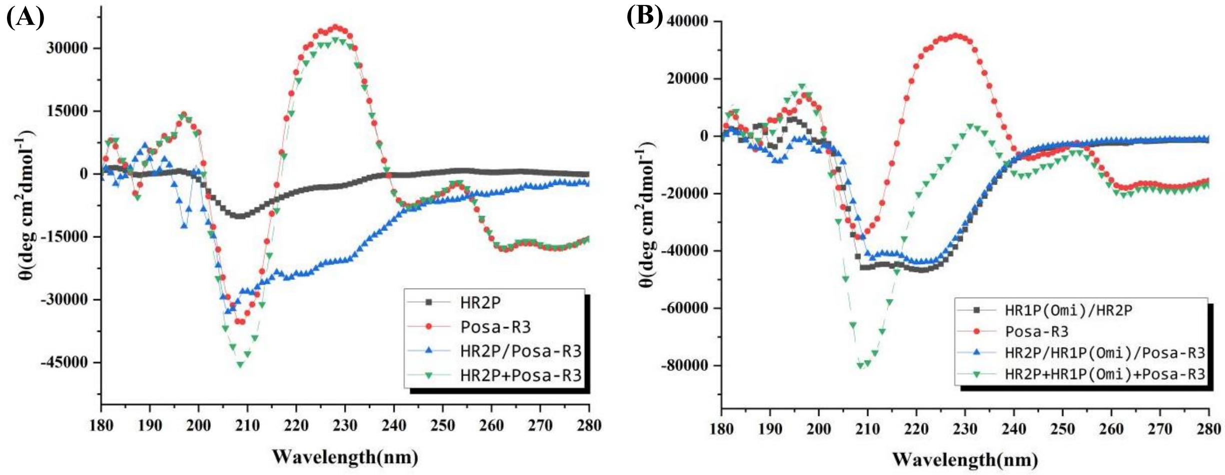

Figure S16. (A) CD spectrum of Posa-R3 and HR2P complex (HR2P/Posa-R3 represents the Posa-R3 and HR2P mixed solution); (B) CD spectrum of Posa-R3 and HR1P(Omi)/HR2P complex (HR2P/HR1P(Omi)/Posa-R3 represents the Posa-R3, HR1P(Omi) and HR2P mixed solution).

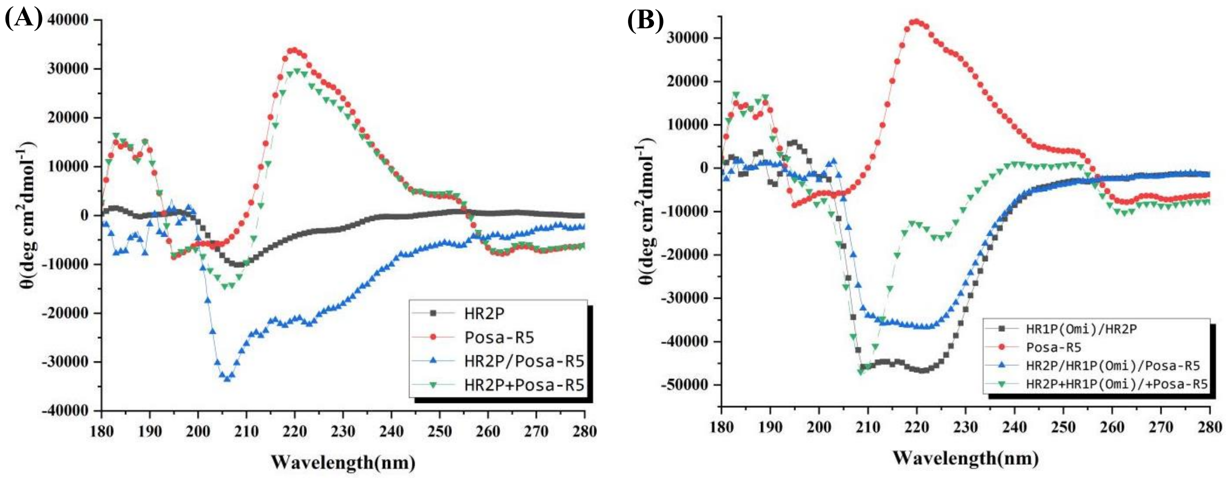

Figure S17. (A) CD spectrum of Posa-R5 and HR2P complex (HR2P/Posa-R5 represents the Posa-R5 and HR2P mixed solution); (B) CD spectrum of Posa-R5 and HR1P(Omi)/HR2P complex (HR2P/HR1P(Omi)/Posa-R5 represents the Posa-R5, HR1P(Omi) and HR2P mixed solution).

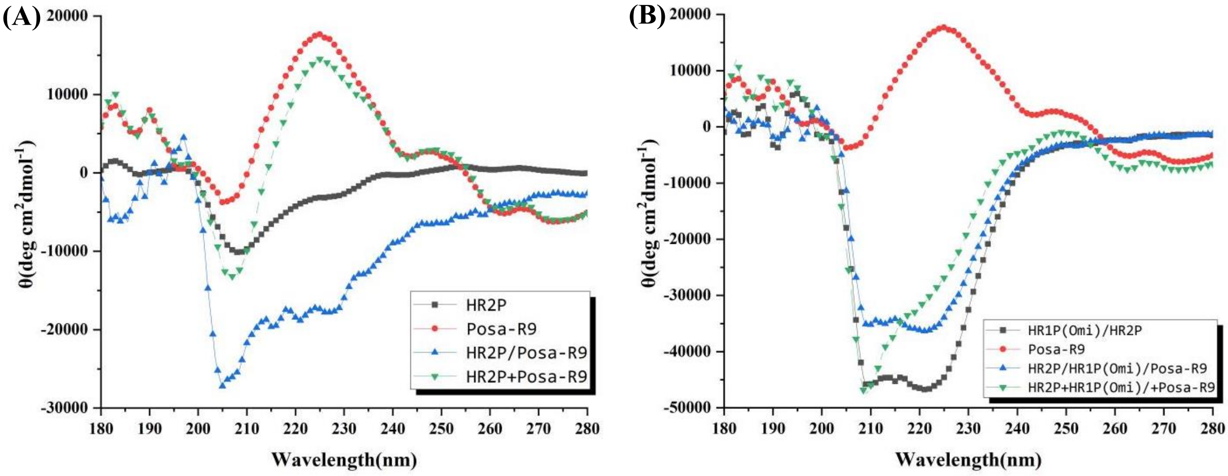

Figure S18. (A) CD spectrum of Posa-R9 and HR2P complex (HR2P/Posa-R9 represents the Posa-R9 and HR2P mixed solution); (B) CD spectrum of Posa-R9 and HR1P(Omi)/HR2P complex (HR2P/HR1P(Omi)/Posa-R9 represents the Posa-R9, HR1P(Omi) and HR2P mixed solution).

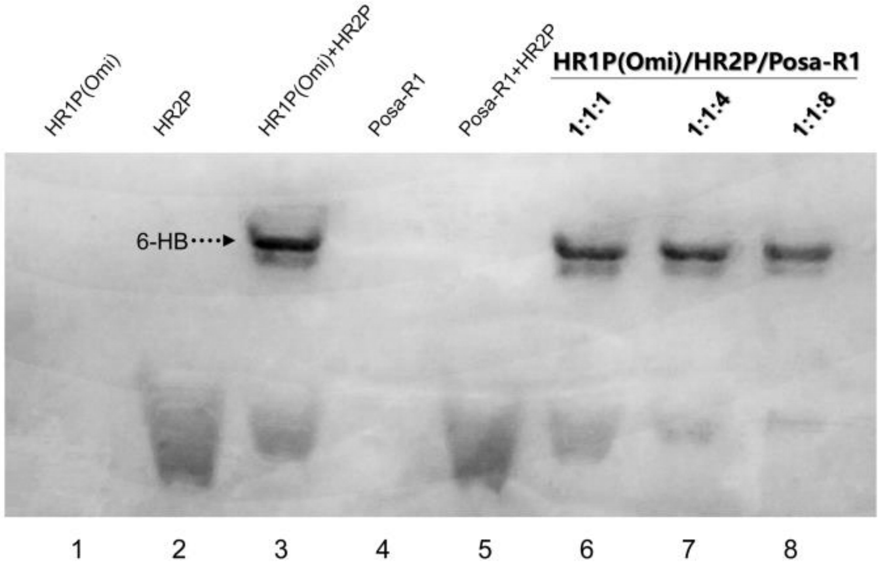

Figure S19. (A) N-PAGE analysis of Posa-R1 with target peptide (HR1P(Omi)/HR2P/Posa-R1 represents the Posa-R1, HR1P(Omi) and HR2P mixed solution)

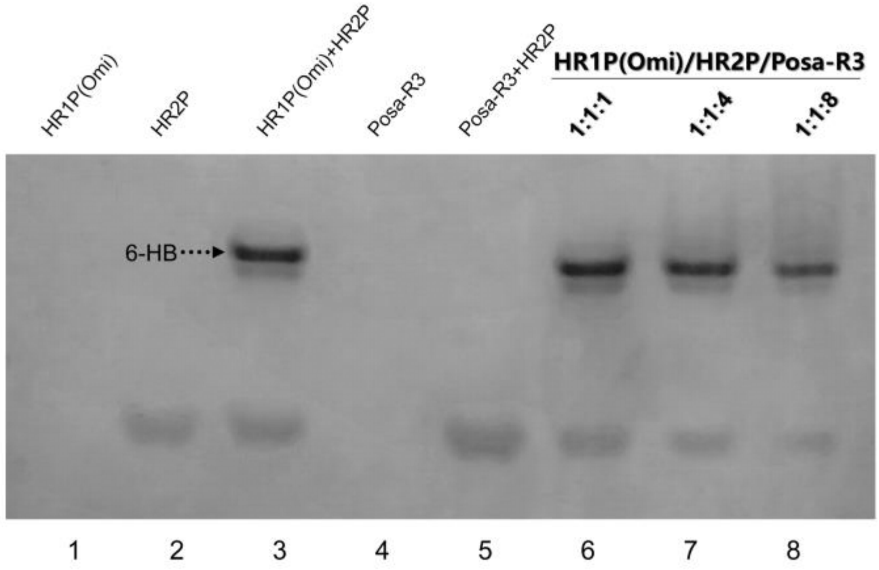

Figure S20. (A) N-PAGE analysis of Posa-R3 with target peptide (HR1P(Omi)/HR2P/Posa-R3 represents the Posa-R3, HR1P(Omi) and HR2P mixed solution)

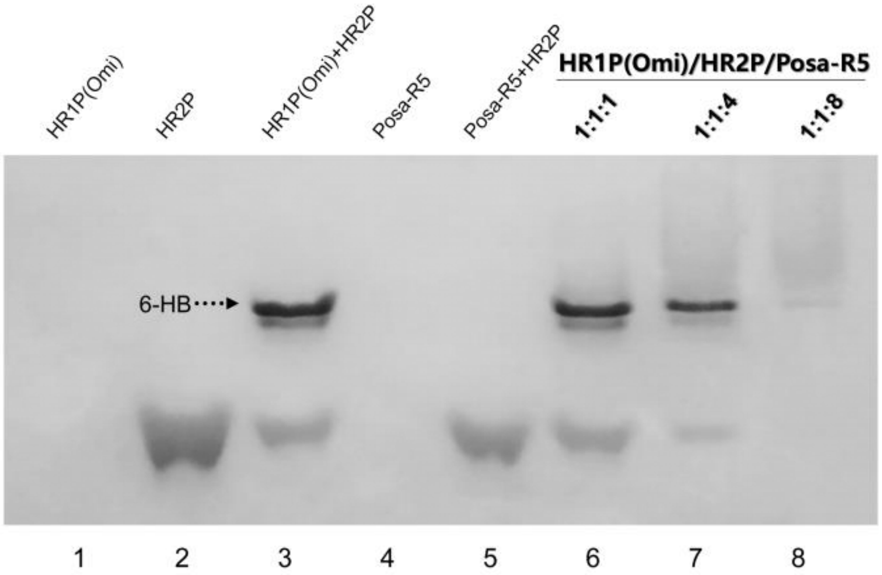

Figure S21. (A) N-PAGE analysis of Posa-R5 with target peptide (HR1P(Omi)/HR2P/Posa-R5 represents the Posa-R5, HR1P(Omi) and HR2P mixed solution)

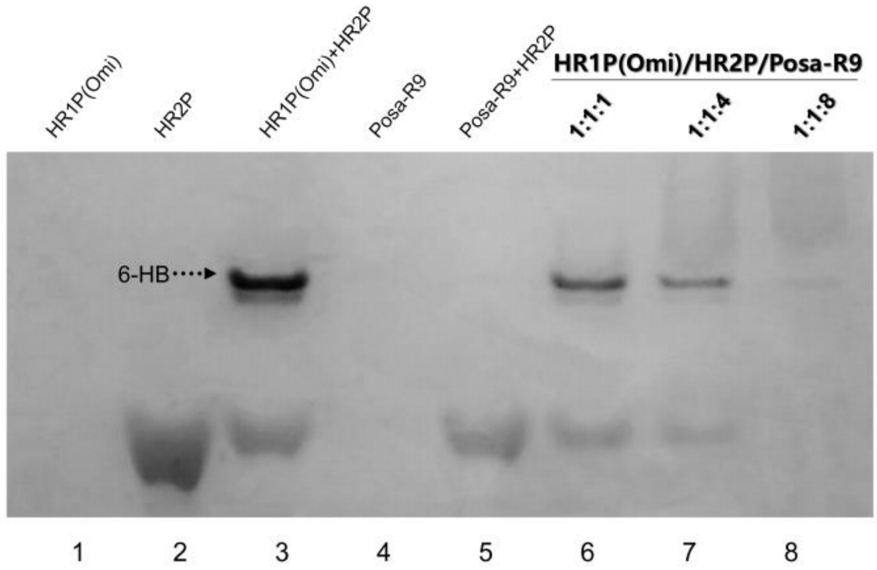

Figure S22. (A) N-PAGE analysis of Posa-R9 with target peptide (HR1P(Omi)/HR2P/Posa-R9 represents the Posa-R9, HR1P(Omi) and HR2P mixed solution)

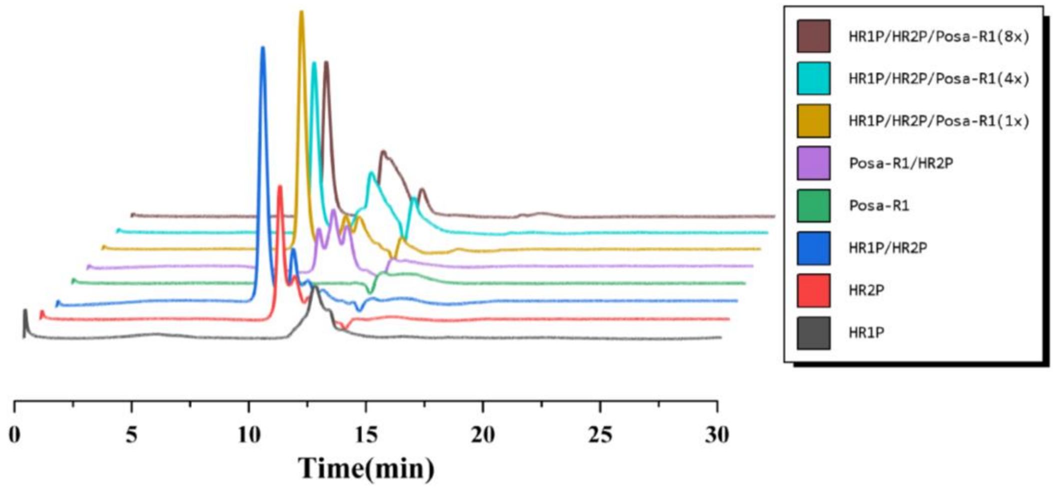

Figure S23. SE-HPLC analysis of Posa-R1 with target peptide (HR1P(Omi)/HR2P/Posa-R1 represents the Posa-R1, HR1P(Omi) and HR2P mixed solution)

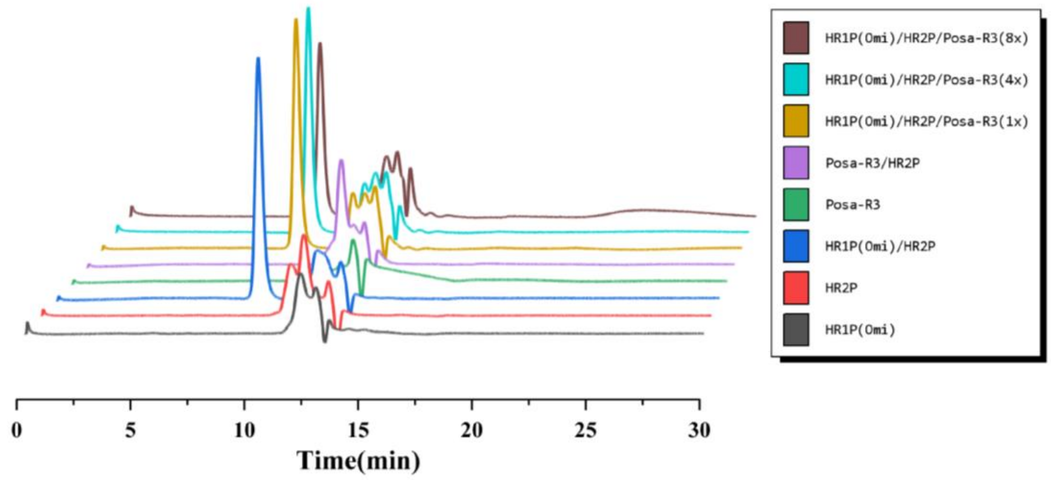

Figure S24. SE-HPLC analysis of Posa-R3 with target peptide (HR1P(Omi)/HR2P/Posa-R3 represents the Posa-R3, HR1P(Omi) and HR2P mixed solution)

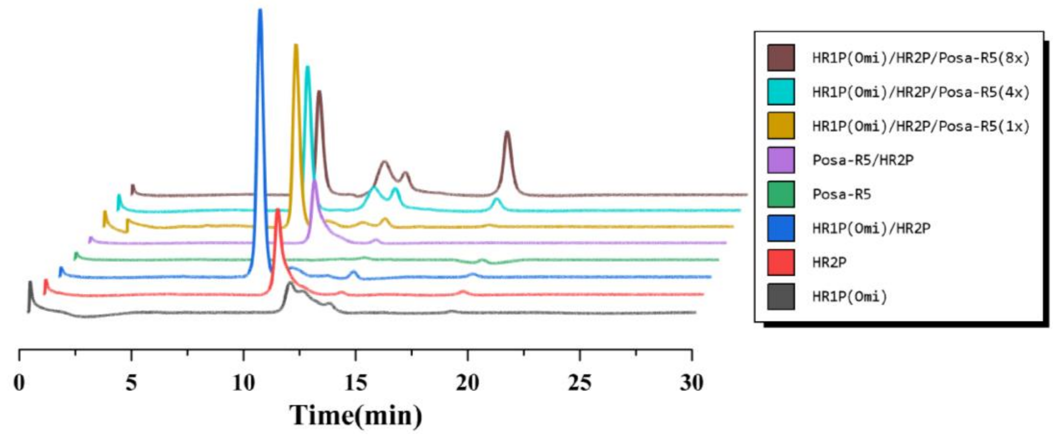

Figure S25. SE-HPLC analysis of Posa-R5 with target peptide (HR1P(Omi)/HR2P/Posa-R5 represents the Posa-R5, HR1P(Omi) and HR2P mixed solution)

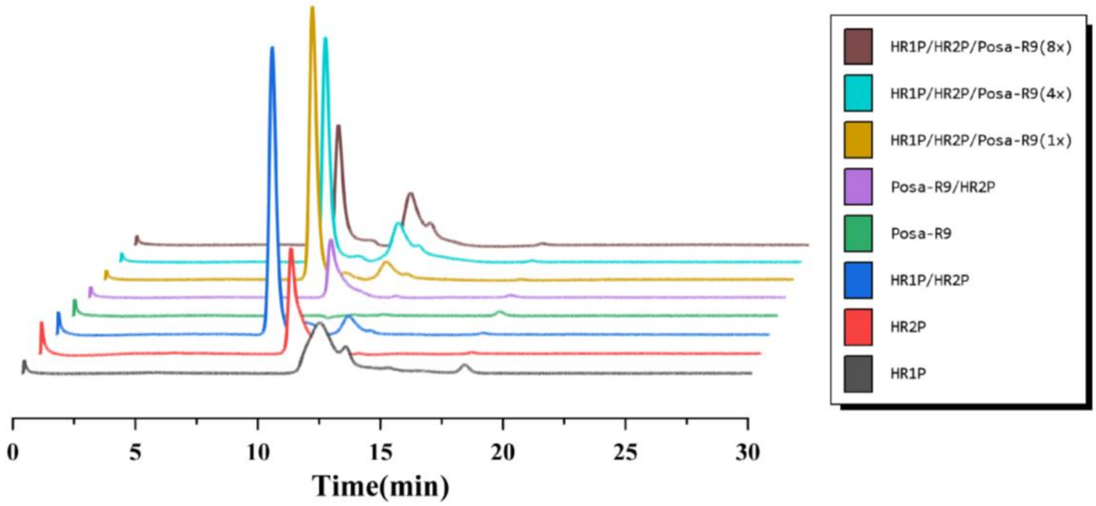

Figure S26. SE-HPLC analysis of Posa-R9 with target peptide (HR1P(Omi)/HR2P/Posa-R9 represents the Posa-R9, HR1P(Omi) and HR2P mixed solution)

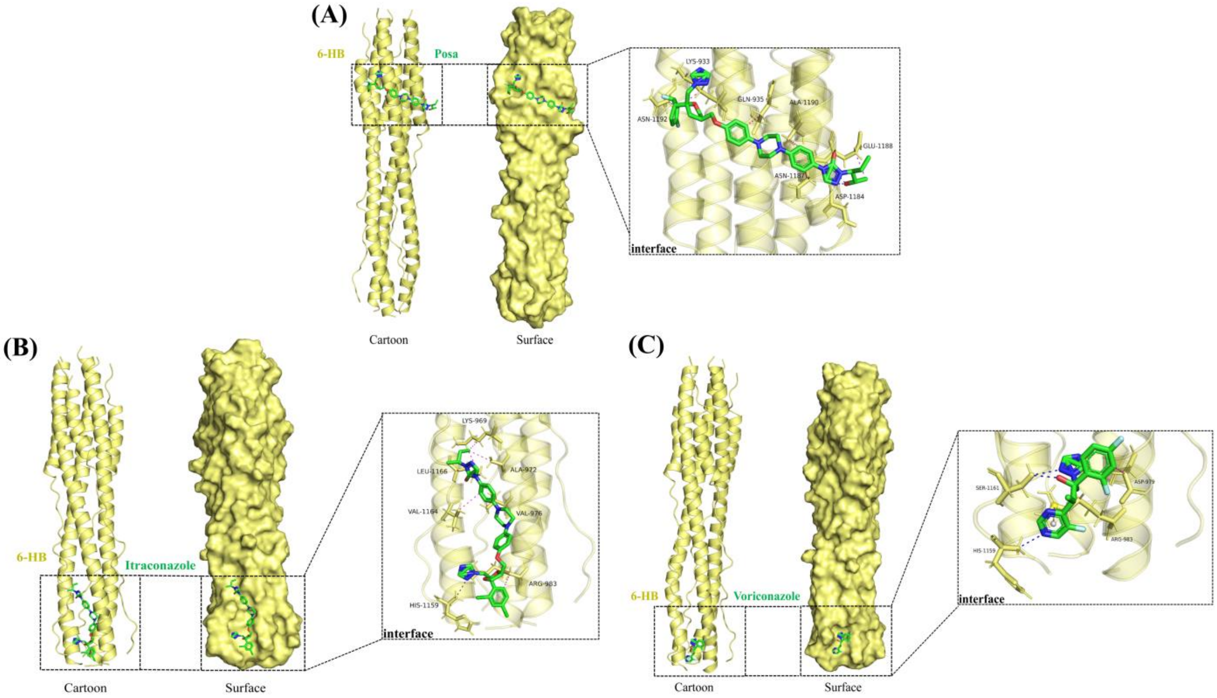

Figure S27. (A) Molecular docking analyses of Posa with the 6-HB region; (B) Molecular docking analyses of Itraconazole with the 6-HB region; (C) Molecular docking analyses of Voriconazole with the 6-HB region. (Important residues are shown as sticks and labeled. The blue dashed lines are the hydrogen bonds, red dashed lines are the salt bridges and magenta dashed lines are the hydrophobic interactions, orange dashed lines are the cation-π interaction)

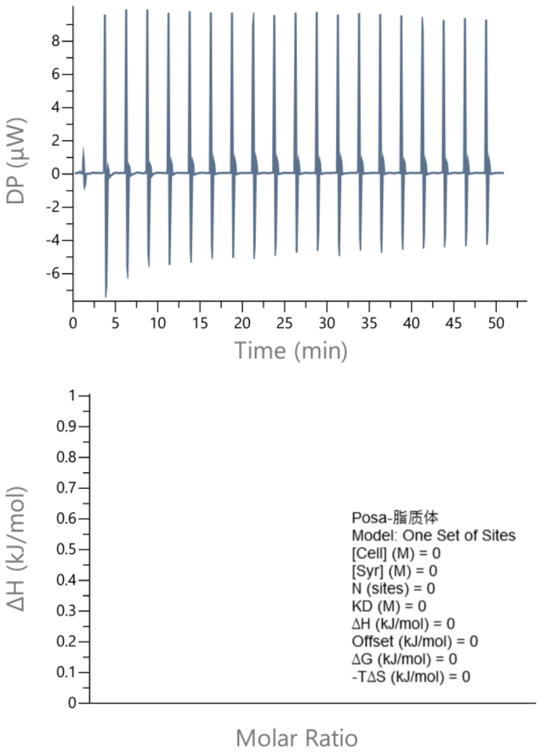

Figure S28. Analysis of the binding of Posa to a lipid bilayer.

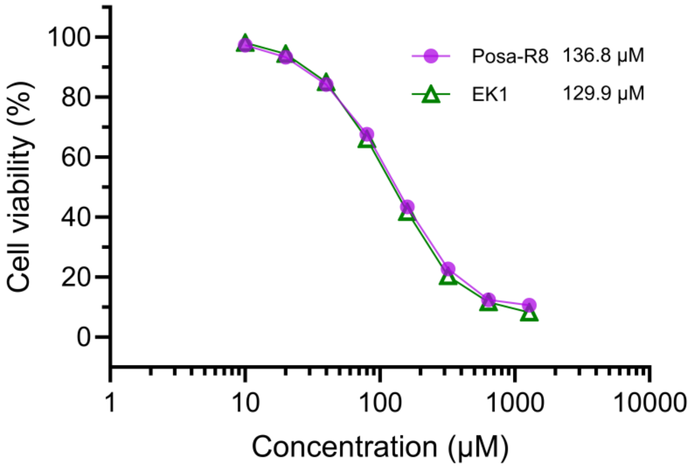

Figure S29. Cytotoxicity diagram of Posa-R8 and EK1.
